# Coordination of the AMPK, Akt, mTOR, and p53 Pathways under Glucose Starvation

**DOI:** 10.3390/ijms232314945

**Published:** 2022-11-29

**Authors:** Yifan Zhou, Feng Liu

**Affiliations:** National Laboratory of Solid State Microstructures, Department of Physics, Collaborative Innovation Center of Advanced Microstructures, and Institute for Brain Sciences, Nanjing University, Nanjing 210093, China

**Keywords:** glucose starvation, cell-fate decision, network modeling, biphasic dynamics, senescence, apoptosis

## Abstract

Glucose is a direct energy source for eukaryotic cells, and its deficiency elicits complex stress responses and diverse cellular outcomes. Although several signaling pathways involved have been identified, how they coordinately dictate the cell fate remains obscure. We propose a minimal network model for the cellular response to glucose restriction, characterizing the glucose uptake and signaling of the AMPK, Akt, mTOR, and p53 pathways. We demonstrate that in the presence of sufficient growth factors and amino acids, cells may undergo proliferation, senescence, or apoptosis, depending on the extracellular glucose level. AMPK is first activated upon glucose limitation, activating p53 to induce cell-cycle arrest; possibly, cells resume proliferation after timely glucose restoration. For long-term energy stress, cell senescence is maintained by low/intermediate levels of p53 and persistent activation of mTOR and Akt, or cells commit apoptosis when the proteins undergo biphasic dynamics, e.g., p53 switches from intermediate levels to high levels while mTOR and Akt become inactivated in the later phase. The biphasic dynamics of p53 are associated with flipping of two bistable switches. Appropriate mTOR levels are required for optimal cell-fate decision. This work suggests that senescence and apoptosis occur sequentially in glucose-depleted cells, and a theoretical framework is provided for exploring the cellular response to energy stress.

## 1. Introduction

To proliferate, living cells grow and divide through the cell cycle, which is under tight control via various checkpoints [1]. Sufficient amounts of adenosine 5′-triphosphate (ATP) are required to sustain cell viability and drive cell cycle progression. There exists an energy checkpoint monitoring the cellular energy status and triggering the cellular response to energy deficiency by engaging multiple signaling pathways [2]. They include the AMPK (adenosine monophosphate (AMP)-activated protein kinase), PI3K (phosphatidylinositol 3-kinase)/Akt, mTOR (mechanistic target of rapamycin), and p53 pathways [3,4,5,6]. Generally speaking, the PI3K/Akt pathway signals the availability of growth factors and stimulates nutrient uptake to support mTOR-dependent cell growth and proliferation, while the AMPK pathway signals the lack of energy and triggers the p53-dependent stress response. How these pathways synergistically guide the cellular response to energy stress has been the subject of intensive research.

Upon glucose starvation, AMPK is first activated as a sensor of cellular energy status [7,8,9,10]. AMPK exists as a heterotrimer, comprising a catalytic (α) and two regulatory (β and γ) subunits; once activated, AMPK stimulates ATP-generating pathways and inhibits energy-consuming pathways to restore energy homeostasis. Activated AMPK also phosphorylates p53 on Ser15, leading to its stabilization and activation [2,11,12]. Active p53 mediates the cellular response primarily as a transcription factor [13]. p53 induces p21 to arrest the cell cycle, promoting cell survival [2]; sustained activation of p53 may lead to cellular senescence (i.e., irreversible cell-cycle arrest) or apoptosis [14,15,16]. Notably, glucose depletion-induced apoptosis is associated with significant accumulation of p53 phosphorylated on Ser46 and transcriptional induction of proapoptotic proteins such as p53AIP1 (p53-regulated apoptosis-inducing protein 1) and PUMA (p53 upregulated modulator of apoptosis) [16,17]; it is also coupled with the inhibition of Akt activity [15]. Nevertheless, how the cell fate is determined after stress and how it depends on the extracellular glucose level remain obscure.

mTOR is the catalytic subunit of two complexes, mTOR complex 1 (mTORC1) and mTORC2, which have distinct physiological functions [6]; here we focus on the former. mTORC1 activity is modulated by upstream factors including growth factors, glucose, and amino acids. Although mTORC1 activity is normally associated with cell growth, active mTOR is essential for cell senescence when the cell cycle is blocked [14,18]. On the other hand, senescence is suppressed when mTOR activity is inhibited by high levels of p53 [19]. It is worthy to explore how the interplay between the p53 and mTOR pathways regulates senescence induction, specifically how the level of p53 tips the balance between cell senescence and apoptosis.

**Figure 1:**
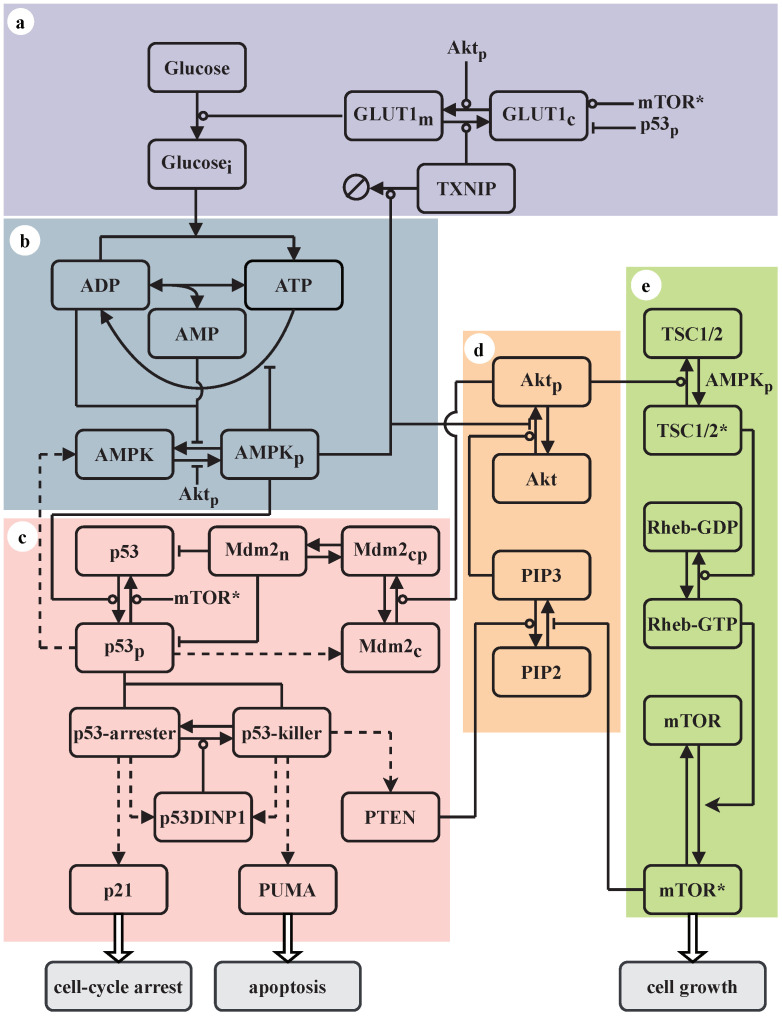
Schematic of the model. The model consists of five modules: glucose uptake (**a**), the AMPK (**b**), p53 (**c**), PI3K/Akt (**d**), and TSC/mTOR (**e**) pathways. State transition is represented by arrow-headed lines, and the promotion and inhibition of state transition are denoted by circle- and bar-headed lines, respectively. The regulation of gene expression by p53 is denoted by dashed lines. p21, PUMA, and activated mTOR (mTOR*) promote cell-cycle arrest, apoptosis, and cell growth, respectively. The input to the system is the extracellular glucose level.

The relationships between the four pathways above are not simply that AMPK and p53 inhibit the Akt and mTOR pathways upon glucose deficiency. Instead, there exists complicated crosstalk between them, and they constitute a network to ensure cells under stress to respond optimally. Thus, it is necessary to elucidate how these pathways are coordinated to dictate the cell fate. Accordingly, a detailed analysis of the dynamics of signaling pathways is indispensable. To this end, we constructed a minimal network model integrating the AMPK, Akt, mTOR, and p53 signaling pathways (Figure 1). Within this context, cell proliferation, senescence, or apoptosis may be elicited, relying on the cellular energy status. We identified the network dynamics associated with each cellular outcome and probed the underlying mechanism for the cell-fate decision among the three alternatives. This study presents an integrated kinetic picture of the cellular response to glucose starvation.

## 2. Results

### 2.1. Network Model

A five-module network model is proposed to characterize the cellular response to glucose starvation (Figure 1). In mammalian cells, extracellular glucose is transported into the cytoplasm via the protein family of glucose transporters (GLUTs). The GLUT family comprises 14 isoforms, among which GLUT1 is the most ubiquitously expressed and responsible for the basal glucose uptake [20]. Glucose uptake is stimulated by enhancing the translocation of GLUT1 from its storage vesicles to the plasma membrane. This process is modulated by Akt and Thioredoxin-interacting protein (TXNIP). Activated Akt (Akt_p_) can facilitate GLUT1 transporter activity and recycling while inhibiting GLUT1 internalization [21], whereas TXNIP represses glucose uptake by binding to GLUT1 and eliciting its internalization [22]. Moreover, GLUT1 production is regulated by various proteins such as p53 and mTORC1; activated p53 (p53_p_) binds the *glut1* promoter to repress its transcription [23], whereas activated mTOR (mTOR*) promotes its expression [24]. GLUT1_m_, GLUT1_c_, and Glucose_i_ are distributed on the plasma membrane and in the cytoplasm, respectively.

Once into the cytoplasm, glucose is processed through glycolysis or oxidative phosphorylation, with ADP converted to ATP; meanwhile, the hydrolysis of ATP into ADP provides energy that supports various physiological processes. The reaction 2ADP↔ATP+AMP is catalyzed by the adenylate kinase [10]. Upon energy stress, the ATP reserve begins to be depleted, leading to a rise in ADP:ATP and AMP:ATP ratios. Consequently, ADP and AMP are exchanged for ATP bound to two of the four potential nucleotide-binding sites in AMPKγ. This promotes the phosphorylation of AMPKα on Thr172 by the tumor suppressor liver kinase B1 (LKB1) and enhances the activation of AMPK [9,25].

As a kinase, activated AMPK (AMPK_p_) plays multiple roles in the cellular response to energy stress. It promotes catabolism and represses ATP-consuming reactions. AMPK_p_ phosphorylates p53 on Ser15, resulting in its stabilization and activation [2,11,12]. Active p53 in turn triggers the production of AMPKβ [26]. Thus, p53 and AMPK positively regulate each other, enclosing a positive feedback loop [27,28]. Meanwhile, AMPK_p_ phosphorylates TXNIP to promote its degradation, thereby relieving the internalization of GLUT1 and enhancing glucose uptake [22]. AMPK_p_ phosphorylates the insulin receptor substrate-1 (IRS-1) on Ser794 to further inhibit the IRS-1/PI3K/Akt signaling [15]. AMPK_p_ phosphorylates and activates TSC2 (tuberous sclerosis complex 2), which forms a complex with its partner TSC1.

As we focus on the cellular response to glucose restriction, sufficient growth factors and amino acids are assumed to be available. Thus, growth factors such as insulin bind to receptor tyrosine kinases, and PI3K gets activated via recruitment to IRS-1, resulting in the conversion from PIP2 (phosphatidylinositol 4,5-biphosphate) to PIP3 (phosphatidylinositol 3,4,5-trisphosphate) [3]. For simplicity, here the IRS-1/PI3K cascade is reflected in the basal phosphorylation rate of PIP2. PIP3 is required for recruitment of Akt to the plasma membrane, where Akt can be activated by phosphorylation on Thr308 and Ser473 [29]. Activated Akt negatively regulates AMPK activation either by suppressing its phosphorylation on Thr172 or by phosphorylating Ser485/491 of AMPKα [30]. Akt_p_ phosphorylates TSC2 at different sites than AMPK_p_ does, inhibiting its GAP (GTPase-activating protein) activity toward Rheb, leading to the activation of Rheb and mTORC1 [3].

When recruited to the lysosomal surface, mTORC1 can be activated by Rheb in its GTP-bound state. In contrast, the activated TSC1/2 complex (TSC1/2*) promotes the conversion from Rheb-GTP to Rheb-GDP, thereby inhibiting mTORC1 activity [31,32]. Activated mTORC1 phosphorylates and activates its substrate S6K1, which directly phosphorylates IRS-1 to prevent activation of the PI3K/Akt pathway [33,34]. mTOR* phosphorylates and activates the α4 subunit of the protein phosphatase 2A (PP2A), which dephosphorylates p53 [35], promoting cell survival. The interplay between the PI3K/Akt and TSC/mTOR signaling underlies a long negative feedback loop, tuning the dynamics of mTORC1 and Akt.

Activated p53 transactivates the *mdm2* gene, while Mdm2 targets p53 for degradation [36]; the p53-Mdm2 negative feedback has a key role in tuning p53 dynamics. Only nuclear p53 is considered here, while three forms of Mdm2 are included, i.e., cytoplasmic dephosphorylated Mdm2 (Mdm2_c_), cytoplasmic phosphorylated Mdm2 (Mdm2_cp_), and nuclear Mdm2 (Mdm2_n_). The Akt-mediated phosphorylation of Mdm2_c_ promotes its nuclear entry [37]. Based on its different phosphorylation status, p53_p_ is further divided into p53-arrester and p53-killer, inducing cell-cycle arrest and apoptosis, respectively [38,39]. p53-arrester refers to p53 primarily phosphorylated on Ser15, whereas p53-killer is p53 further phosphorylated on Ser46. p53-arrester transactivates *p21* and *p53dinp1* (p53-dependent damage inducible nuclear protein 1), whereas p53-killer transactivates *p53dinp1*, *puma* and *pten* (phosphatase and tensin homolog) [26,39]. The conversion between p53-arrester and p53-killer is controlled by p53DINP1 [40]. The lipid phosphatase PTEN promotes the transition from PIP3 to PIP2, thereby repressing Akt activation [41]. Thus, there exists a double-negative feedback loop involving p53-killer, PTEN, Akt, and Mdm2. For simplicity, we assume that the activation of p21 and PUMA is considered the marker for cell-cycle arrest and apoptosis, respectively. That is, we did not model the events following the induction of p21 and PUMA, and thus the irreversibility of senescence and apoptosis was not explored.

### 2.2. AMPK as a Sensor of Cellular Energy Status

**Figure 2:**
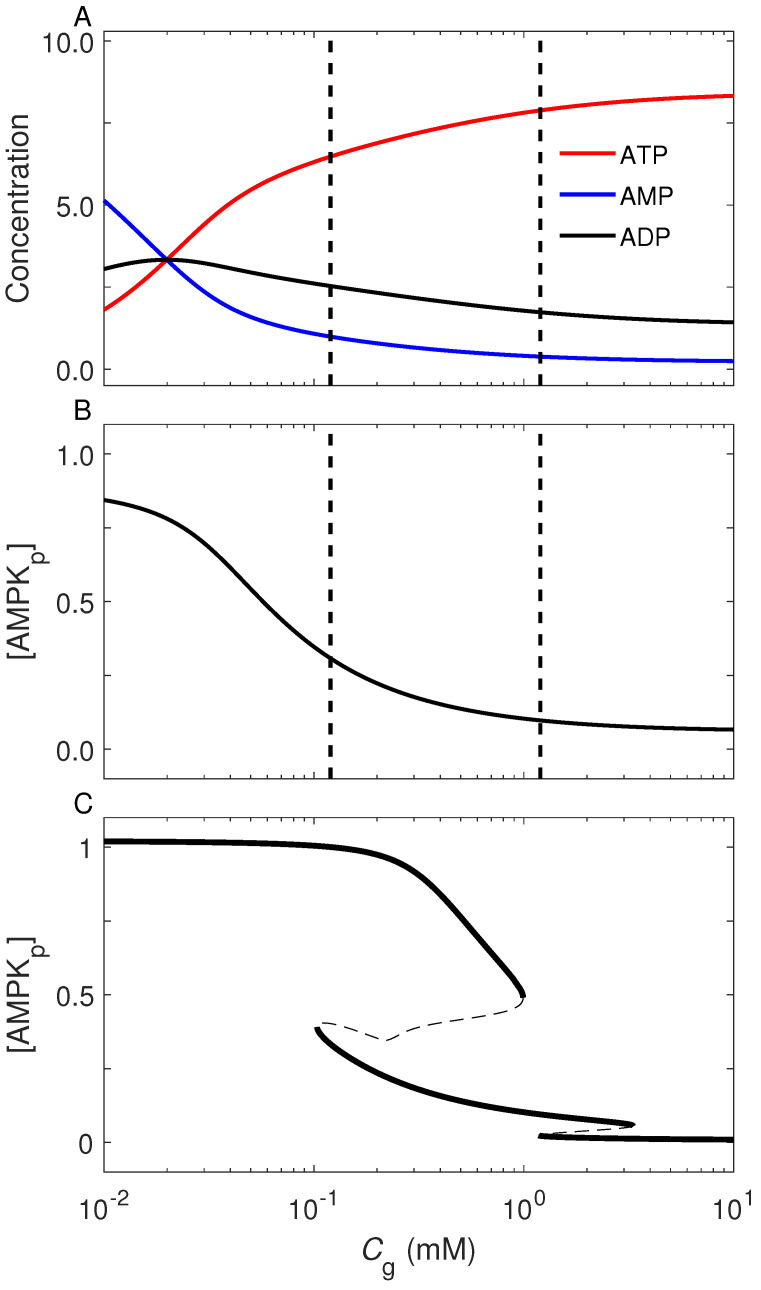
AMPK acts as a sensor of cellular energy status. (**A**,**B**) Steady-state levels of ATP, ADP, and AMP (**A**) and activated AMPK (**B**) vs. the concentration of extracellular glucose, *C*_g_. Here, only modules in Figure 1a,b were considered; the total level of AMPK was set to 1, while the concentrations of Akt_p_, p53_p_ and mTOR* were set to 1, 0.7, and 1, respectively, irrespective of *C*_g_. Two dashed lines mark the lower bifurcation points in panel (**C**). (**C**) Bifurcation diagram for [AMPK_p_] as a function of *C*_g_ in the full model. The stable and unstable steady states are denoted by solid and dashed lines, respectively.

We first explored how AMPK activation is correlated with the cellular energy status, as reflected in relative concentrations of nucleotides. To this end, only modules in Figure 1a,b are simulated, and the feedback modulation from the other modules is replaced by setting the concentrations of involved proteins (i.e., Akt_p_, mTOR* and p53_p_) to be constant, irrespective of the extracellular glucose concentration *C*_g_. Figure 2A shows the steady-state levels of ATP, ADP, and AMP versus *C*_g_. For *C*_g_ > 0.1 mM, the ATP level is far higher than the ADP and AMP levels, and the ATP and AMP levels decrease and increase, respectively, with reducing *C*_g_. The ADP level always exceeds the AMP level unless *C*_g_ is sufficiently small. Under low-glucose conditions, [AMP] is much higher than [ATP].

The steady-state level of AMPK_p_ monotonically rises toward saturation with decreasing *C*_g_ (Figure 2B). AMPK is partially activated at moderate glucose levels, and is fully activated at low levels. This is consistent with the experimental findings [8,9]. Upon glucose limitation, reduced ATP production induces a rise in ADP concentration, and binding of ADP to AMPKγ prevents the dephosphorylation of AMPKα on Thr172. If extracellular glucose is almost deprived, more AMP is produced, and allosteric activation induced by AMP binding to AMPKγ further amplifies AMPK activity. Such a regulatory mode allows AMPK to sense energy deficiency over a wide range. It is worth noting that the bifurcation diagram for [AMPK_p_] in the full model is more complicated (Figure 2C), because the interplay between the four signaling pathways exerts a large influence. There exist three stable branches with basal, low/intermediate, or high levels of [AMPK_p_], separately corresponding to abundant, limited, and deleted glucose. The underlying mechanism will be illuminated later. Together, the concentration of AMPK_p_ is a good gauge of extracellular glucose level.

### 2.3. Cellular Outcome under Glucose Restriction

**Figure 3:**
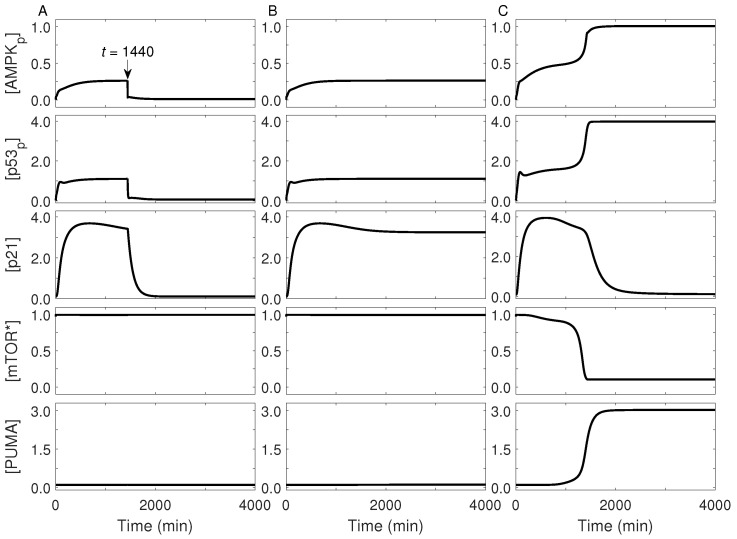
Dynamics of key proteins under typical stress conditions. Shown are time courses of the levels of AMPK_p_, p53_p_, p21, mTOR*, and PUMA (from top to bottom). (**A**) *C*_g_ equals 0.2 mM during the initial 1440 min and 5 mM thereafter. *C*_g_ always equals 0.2 mM (**B**) or 0.08 mM (**C**). The arrow in panel (**A**) indicates the timing when the glucose level is restored to 5 mM.

As growth factors and amino acids are assumed to be abundant, Akt and mTOR are activated under normal energy conditions. Here we investigated how the fate of a proliferating cell varies with glucose restriction. Figure 3 displays the dynamics of key proteins under diverse energy conditions. For limited glucose (e.g., *C*_g_ = 0.2 mM), AMPK is partially activated and p53 is further activated by AMPK_p_, with [AMPK_p_] and [p53_p_] at low or intermediate levels (Figure 3A). p21 is induced by p53 to arrest the cell cycle, while mTOR remains active. If the concentration of extracellular glucose is restored to 5 mM at 24 h, as performed in the experiment [2], then the concentrations of AMPK_p_, p53_p_, and p21 return to their basal levels after a short period. Consequently, the cell should recover to normal proliferation, which agrees well with the finding that temporary glucose restriction induces a reversible cell-cycle arrest while mTOR is kept active [2].

If extracellular glucose is limited persistently, both [AMPK_p_] and [p53_p_] are kept at low or intermediate levels (Figure 3B). Accordingly, p21 is induced persistently, leading to long-lasting cell-cycle arrest; meanwhile, mTOR remains activated. In this case, senescence is evoked so that the cell can survive the moderate energy stress. Our results are in agreement with several experimental observations: both cell-cycle arrest and active mTOR are required for induction of senescence [18], moderate levels of p53 allow for senescence [19,42], and AMPK activation is maintained during senescence [43].

Upon glucose deprivation (e.g., *C*_g_ = 0.08 mM), the cell first undergoes cell-cycle arrest, since AMPK is partially activated and p21 is induced; around 1600 min, AMPK becomes fully activated and [p53_p_] jumps to a high level, whereas [p21] gradually drops to its basal level and mTOR becomes inactive (Figure 3C). Meanwhile, PUMA is induced to trigger cell apoptosis, with its level rising dramatically. In this case, the protein dynamics exhibit biphasic behavior. mTOR activity is repressed greatly in the second phase, consistent with the finding that mTOR is inhibited following acute glucose starvation [31]. The timing for apoptosis induction by high levels of p53 phosphorylated on Ser46 coincides with the experimental data [16]. Together, there may exist three kinds of cellular outcome after glucose restriction: proliferation, senescence, and apoptosis.

**Figure 4:**
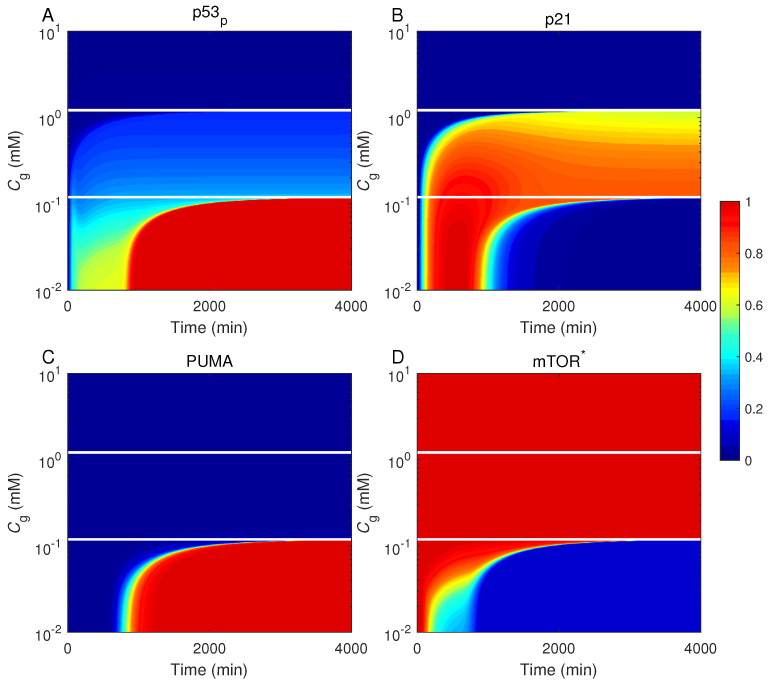
Different cellular outcomes after glucose restriction. Displayed are the levels of p53_p_ (**A**), p21 (**B**), PUMA (**C**), and mTOR* (**D**) as a function of time and glucose level *C*_g_. The protein levels are normalized to their respective peak values (see the color scale bar on the right). There exist three kinds of cellular outcomes: proliferation (*C*_g_ ≥ 1.2 mM), senescence (0.12 ≤ *C*_g_ < 1.2 mM), and apoptosis (*C*_g_ < 0.12 mM). Two horizontal lines mark *C*_g_ = 0.12 and 1.2 mM.

To further probe the cell-fate decision systematically, we showed the levels of p53_p_, p21, mTOR*, and PUMA as a function of time and *C*_g_ in Figure 4. The extracellular glucose level is separately designated adequate, limited, and deprived on three intervals separated by two horizontal lines (at 1.2 mM and 0.12 mM). In the presence of adequate glucose, p53_p_, p21, and PUMA remain at basal levels, whereas mTOR is active persistently. This indicates that the cell undergoes normal proliferation. With a limited amount of glucose, p53_p_ and PUMA remain at intermediate and basal levels, respectively, whereas p21 is kept at high levels. Meanwhile, mTOR activation is maintained. Thus, the cell becomes senescent under prolonged glucose-limiting conditions. Once extracellular glucose is deprived, p53_p_ undergoes the biphasic dynamics, with its concentration switching from intermediate levels in the first phase to high levels in the second phase. p21 and PUMA are primarily induced in the first and the second phase, respectively, while mTOR is deactivated in the second phase. Thus, the cell commits apoptosis in the later phase. Collectively, the cell fate is closely associated with the network dynamics. Specifically, both the level and transcriptional activity of p53 are tightly regulated to trigger distinct cell outcomes.

To examine whether or not the conclusions above depend heavily on parameter values, we performed a series of parameter sensitivity analysis. First, for each of the 107 parameters, its value is increased or decreased relative to its default value *p*_d_, while the others are kept at default values. If plausible cellular outcome can be evoked at diverse glucose concentrations (e.g., three cell fates can be differentiated, proliferation should not occur for *C*_g_ < 0.1 mM, and apoptosis should not emerge for *C*_g_ > 2 mM), then this parameter value is considered reasonable. In this manner, we got the upper and lower bounds for this parameter, *p*_U_ and *p*_L_, and then calculated *p*_U_/*p*_d_ and *p*_L_/*p*_d_, which are shown in Figure S1A. Some parameters can even take ten-fold or tenth their default values. The network can behave normally even when any parameter is decreased by 40% or increased by 80%. Figure S1B displays the ratios for eight most sensitive parameters, including *k*_pp53_ (AMPK-dependent phosphorylation rate of p53) and AXP_t_ (total concentration of ATP, ADP and AMP); obviously, these quantities markedly affect the network dynamics. These results suggest that the network can function normally in a relatively wide parameter range.

Second, we explored the responses of a population of 2000 isogenic cells to the same stress signal. Given the cellular heterogeneity, every parameter value for each cell is randomly chosen from 85% to 115% of its default value. At *C*_g_ = 0.2 mM, although the dynamics of [p53_p_] exhibit marked variability among the cells, their steady-state concentrations are centered around basal, low/intermediate, or high levels, and the one- or two-step dynamics are involved (Figure S2). Figure S3 shows the steady-state levels of p21, PUMA, and mTOR* versus the p53_p_ level; the data points cluster locally without overlap, and three sets in each panel correspond to different cell fates. At *C*_g_ = 2 mM, the steady-state value of [p53_p_] is still centered around the basal or intermediate levels (Figure S4), and two data sets are well separated in Figure S5; in this case, no apoptosis can be induced. These results definitely indicate that each cellular outcome is uniquely associated with the specific network dynamics, i.e., relative expression levels of proteins. These results also suggest that our model and parameter setting are robust in accounting for the choice among three alternatives. Together, a reliable cell-fate decision can be made after energy stress provided that the signaling pathways involved are coordinated to control the protein levels within appropriate ranges.

### 2.4. Cell-Fate Decision Mediated by p53

**Figure 5:**
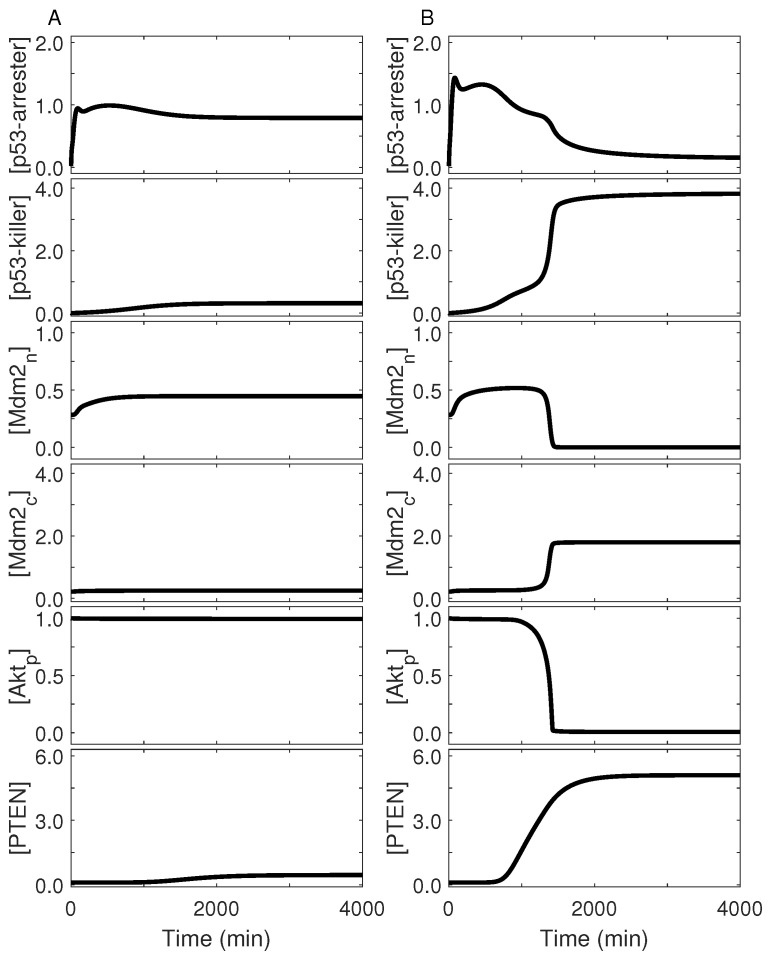
p53 dynamics and the associated feedback control. Shown is temporal evolution of the levels of p53-arrester, p53-killer, nuclear Mdm2, cytoplasmic dephosphorylated Mdm2, Akt_p_, and PTEN (from top to bottom) at *C*_g_ = 0.2 mM (**A**) or 0.08 mM (**B**).

We further probed the underlying mechanism for p53 dynamics in terms of feedback loops centered on p53. Upon glucose limitation (e.g., *C*_g_ = 0.2 mM), activated AMPK phosphorylates p53 on Ser15, and active p53 in turn induces the synthesis of AMPKγ. p53-arrester is the dominant form of p53_p_, and thus PTEN is present only at basal levels (Figure 5A). Akt remains active, phosphorylating Mdm2_c_ to promote its nuclear entry. Because [Mdm2_n_] is markedly greater than zero and active mTORC1 dephosphorylates p53_p_ via PP2A, p53_p_ is kept at low levels. Thus, the p53-Mdm2 and AMPK-p53 feedback loops are mainly responsible for the one-step dynamics of p53_p_. If extracellular glucose is deprived (e.g., *C*_g_ = 0.08 mM), activation of AMPK is enhanced, and the level of p53-arrester is elevated (Figure 5B). The inhibition of Akt and mTOR by AMPK is also enhanced. The accumulation of p53DINP1 promotes the phosphorylation of p53 on Ser46, contributing to the induction of PTEN. PTEN represses Akt activity, further leading to inhibition of mTOR activity and nuclear entry of Mdm2. Thus, the degradation of p53 by Mdm2_n_ is remarkably suppressed (i.e., the p53-Mdm2 negative feedback is weakened), whereas the p53-PTEN-Akt-Mdm2 positive feedback is strengthened. Consequently, [p53_p_] is driven to high levels, and p53-killer is absolutely predominant over p53-arrester. Thus, the biphasic dynamics of p53_p_ result from the alternate predominance of distinct feedback loops. Of note, p53 also exhibits biphasic dynamics in the cellular response to severe DNA damage [39].

**Figure 6:**
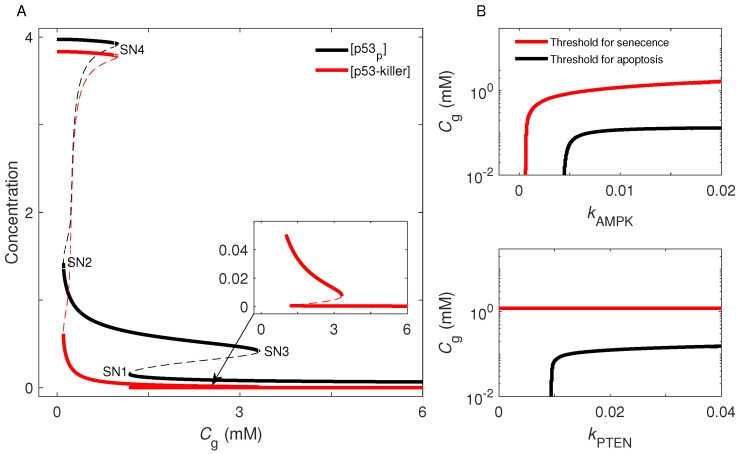
Bistable switches underlying p53 dynamics. (**A**) Bifurcation diagrams for the levels of p53_p_ and p53-killer as a function of *C*_g_. The stable and unstable steady states are denoted by solid and dashed lines, respectively. The inset is an enlarged view of the diagram for [p53-killer]. (**B**) Dependence of the thresholds of *C*_g_ for induction of apoptosis and senescence on the p53-inducible synthesis rates of AMPK and PTEN, *k*_AMPK_ (**upper**) and *k*_PTEN_ (**lower**).

It is well-known that a motif composed of coupled negative and positive feedback loops or double positive ones can behave as a bistable switch [44,45]. Indeed, here the p53 dynamics are governed by two bistable switches, and the determination of cell fate is associated with the flipping of switches. The first switch mainly originates from the p53-Mdm2 and AMPK-p53 loops, while the second switch is closely associated with the AMPK-p53 and p53-PTEN-Akt-Mdm2 loops. Figure 6A displays the dependence of the steady-state levels of p53_p_ and p53-killer on *C*_g_. In the bifurcation diagram for p53_p_, there exist four saddle-node (SN) bifurcation points (SN1–4), at which the corresponding glucose level is denoted by *C*_gi_ (*i* = 1–4). Given that p53_p_ is always kept at basal levels for *C*_g_ > *C*_g3_ (3.3 mM), decreasing *C*_g_ until *C*_g1_ (1.2 mM) corresponds to moving leftwards along the lowest branch. When *C*_g_ is decreased from *C*_g1_ to *C*_g2_ (0.12 mM), [p53_p_] rises along the middle stable branch. If *C*_g_ < *C*_g2_, p53_p_ will be kept at high levels. For p53-killer, there also exist four saddle-node bifurcation points, and only when *C*_g_ < *C*_g2_ can [p53-killer] switch from a low level to high level. Therefore, two transitions occur at SN1 and SN2 (with decreasing *C*_g_), corresponding to the induction of senescence and apoptosis, respectively, and *C*_g1_ and *C*_g2_ denote the corresponding threshold concentrations of extracellular glucose. Moreover, the hysteresis feature of a bistable switch naturally ensures that the state transition is robust against small fluctuations in glucose levels. Collectively, the cell proliferates under adequate glucose; upon glucose deficiency, a reliable cell-fate decision is made through turning on the first switch to induce senescence, or further turning on the second switch to trigger apoptosis. That is, a three-fate decision can be robustly made in this manner.

Furthermore, we investigated the influence of AMPK-p53 and p53-PTEN-Akt-Mdm2 loops on cell-fate decision. For simplicity, the p53-inducible synthesis rates of AMPK and PTEN, *k*_AMPK_ and *k*_PTEN_, are chosen to characterize the feedback strength, respectively. Figure 6B shows the dependence of *C*_g1_ and *C*_g2_ on *k*_AMPK_ and *k*_PTEN_. Notably, AMPK is indispensable for the induction of senescence or apoptosis. Blocking the p53-mediated induction of AMPKβ (i.e., *k*_AMPK_ = 0) desensitizes the system; p53 is largely inactivated, even in response to glucose depletion (Figure S6). By contrast, only the threshold for apoptosis induction is regulated by the p53-PTEN-Akt-Mdm2 loop. Interrupting the p53-dependent expression of *pten* (i.e., *k*_PTEN_ = 0) does not affect the activation of p53 and induction of senescence, but p53-arrester cannot be converted to p53-killer under glucose starvation. That is, loss of PTEN induces senescence rather than apoptosis even in response to severe stress, consistent with the experimental finding that the PTEN status switches the cell fate between premature senescence and apoptosis [46]. Therefore, appropriate levels of AMPK and PTEN are critical for a balance between different cellular outcomes.

### 2.5. Role for mTOR in Cell-Fate Decision

**Figure 7:**
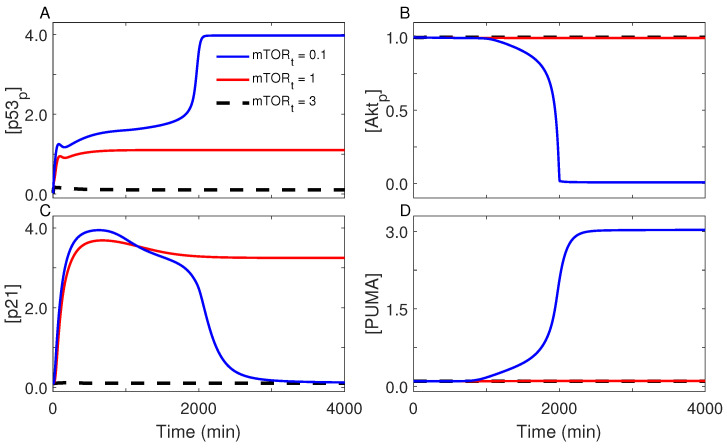
Role for mTOR in cell-fate decision. Shown are time courses of the levels of p53_p_ (**A**), Akt_p_ (**B**), p21 (**C**), and PUMA (**D**) at *C*_g_ = 0.2 mM for mTOR_t_ = 3 (dashed), 1 (red), or 0.1 (blue).

We have demonstrated that, when the cell cycle is arrested, activation of mTOR is essential for the establishment of cell senescence. Here, we further explored the role for mTOR in regulating senescence by changing its total level, mTOR_t_. With the default parameter setting (mTOR_t_ = 1), the cell becomes senescent at *C*_g_ = 0.2 mM; p53_p_ is kept at moderate levels, inducing p21 rather than PUMA, while Akt remains active (Figure 7). At *C*_g_ = 0.2 mM, decreasing mTOR_t_ tends to promote the induction of apoptosis. For mTOR_t_ = 0.1, p53_p_ undergoes the biphasic dynamics, and PUMA is induced in the late phase to trigger apoptosis. This suggests that appropriate levels of activated mTOR are a prerequisite for senescence induction. Additionally, the mTOR inhibitor rapamycin may be used to inhibit the induction of senescence. Conversely, increasing mTOR_t_ contributes to cell survival. For mTOR_t_ = 3, p53 is totally inactive, and the cell undergoes proliferation despite limited glucose availability, possibly leading to tumorigenesis. Indeed, overactivation of growth-promoting pathways and loss of cell-cycle arrest were typically observed in tumor cells [47]. Therefore, mTOR also has a key role in guiding cell fate after energy stress, and its concentration should be controlled within an appropriate range to guarantee a reasonable choice of cell fate.

## 3. Discussion

It is well established that there exists an energy checkpoint at the G1/S boundary [2]. Here, new insights are gained into how the information about the intracellular energy status is processed. Our results suggest a two-step mechanism for the cellular response to energy stress. The first step is to decide whether sufficient energy is maintained to complete the cell cycle. If yes, the cell continues to proliferate; otherwise, both AMPK and p53 are activated, and the cell cycle is arrested to switch off some energy-consuming processes and provide time for energy restoration. If the energy restriction is long lasting, then the second step is to determine whether energy levels are sufficient to sustain cell viability. If yes, the cell becomes senescent; otherwise, survival signals are inhibited and proapoptotic genes are induced to trigger apoptosis. That is, even when the cell is exposed to glucose deprivation, apoptosis will not be induced soon after stress. This seems reasonable: in contrast to DNA damage, energy stress more probably requires an adaptive response since simple restoration of nutrients can rapidly restore a starved cell, and only sustained severe stress requires eliciting an apoptotic response. This two-step control mode is advantageous to cell survival; limited energy is saved to maintain cell viability, thereby avoiding unnecessary cell death.

Here, three cell fates are represented by distinct attractor states of the network, and their transitions are associated with two bistable switches, which are mainly governed by the AMPK-p53 and p53-Mdm2 feedback loops and the AMPK-p53 and p53-PTEN-Akt-Mdm2 loops, respectively. In the presence of adequate glucose, neither of the switches is turned on, and p53 remains at basal levels. In response to limited glucose availability, the first switch is turned on, and thus p53 is kept at low or intermediate levels. If extracellular glucose is deprived, the first and the second switch are flipped sequentially, with p53 driven to high levels in the later phase. This dual-switch mode provides a robust mechanism for a three-state choice. Indeed, similar mechanisms are responsible for cellular signaling such as epithelial-to-mesenchymal transition [48].

Although we have built an integrated network to depict a coherent picture of cellular response, multiple aspects of cellular singling are not covered here but worth investigating, such as autophagy [6,49,50]. It would also be interesting to extend the current model by incorporating more effectors of mTORC1 and AMPK, so that more insights could be gained.

The development of senescence is a complicated process, which begins with cell-cycle arrest and ends with fully irreversible senescence phenotype. For simplicity, here we took both the persistent induction of p21 and activation of mTOR as the indicator of senescence. Consistently, experimental results also indicated that the induction of cell senescence is associated with low levels of p53 and activation of mTOR; p53 induces p21 to trigger cell-cycle arrest, while mTOR promotes cell growth [14,18]. It would be intriguing to explore the irreversibility of senescence by further including the downstream targets of pathways that regulate cell-cycle arrest and cell growth.

Our results suggest that AMPK can play dual roles: one as the sensor of low-energy status, and the other as a regulator of cell cycle progression. Since AMPK is involved in the regulation of both senescence and apoptosis, it would be interesting to systematically explore its role in tumor suppression. We also demonstrate that p53 behaves as a central mediator of the cellular response to energy stress, inducing cell-cycle arrest, senescence, or apoptosis, which are the key mechanisms by which p53 suppresses tumor formation [4,51]. On the other hand, tumor cells adapt to distinct energy metabolism, e.g., favoring aerobic glycolysis over oxidative phosphorylation even in the presence of sufficient oxygen [52]. Thus, a deeper understanding of the relationship between metabolism, proliferation, and tumorigenesis may help improve the efficacy of cancer therapies.

## 4. Materials and Methods

As in previous modeling studies [38,39], the concentration of each species in the network (denoted by […]) is represented by a dimensionless state variable in rate equations, while the concentration of extracellular glucose (*C*_g_), which is in units of mM, is considered an input to the network. These ordinary differential equations (ODEs) are presented in Supplementary Materials. The definition of each variable and parameter values are listed in Supplementary Tables S1 and S2, respectively. In our model, the regulation of protein (de)phosphorylation is assumed to follow the Michaelis-Menten kinetics. Besides the basal expression, p53-regulated expression of genes is characterized by Hill function. Each species is assumed to degrade at a rate that is proportional to its concentration. The total levels of Akt, PIP, TSC1/2, Rheb, and mTOR (i.e., those proteins involved in the PI3K/Akt and TSC/mTOR cascades) are separately assumed to be constant, since no remarkable variations were experimentally observed during the stress response [15,31]. The initial concentration of each species was set to its steady-state value at adequate glucose concentrations (i.e., 10 mM). Note that steady-state concentrations of proteins were obtained by setting the right-hand sides of ODEs to zero. The bifurcation diagrams were plotted using Oscill8.

Owing to limited single-cell data availability, parameter values were estimated using a trial-and-error method such that simulation results could be qualitatively consistent with experimental observations. That is, the parameter setting should be subject to some constraints, such as the time taken to trigger apoptosis, the glucose concentration below which apoptosis can be evoked, realization of cell-fate decision, and plausible expression levels of proteins associated with each cellular outcome. Moreover, the production rate and basal degradation rate of p53 are relatively stable under a wide variety of stress conditions, and some interactions within the minimal p53 subnetwork mediating the decision between cell-cycle arrest and apoptosis are relatively fixed (for our purpose here); thus, we also consulted Ref. [39] for parameter choice.

## 5. Conclusions

**Figure 8:**
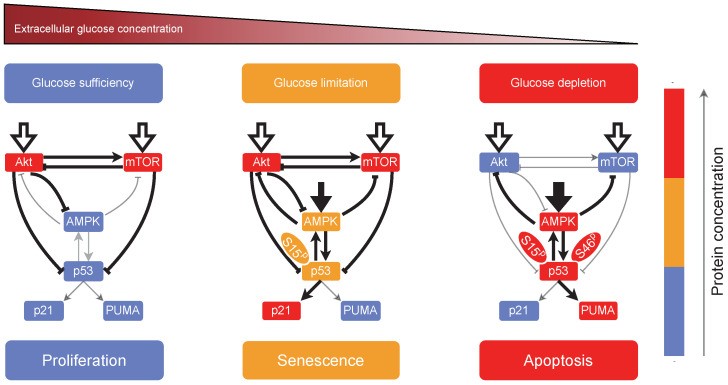
Schematic depiction of cell-fate decision. Cell proliferation, senescence or apoptosis can be induced by distinct combinations of protein activity. Hollow and solid arrows denote the activation from growth factors (amino acids) and glucose starvation, respectively. Arrow- and bar-headed lines separately denote the (in)direct promotion and inhibition between proteins, and grey lines label weak interactions. The color of small boxes marks the level of protein concentration. S15^P^ and S46^P^ separately refer to the phosphorylation of p53 on Ser15 and Ser46.

The current study demonstrated how the AMPK, PI3K/Akt, mTOR, and p53 pathways coordinately determine cellular outcome (Figure 8). AMPK acts as a sensor of cellular energy status and transmits the stress signal by phosphorylating p53, TSC2, IRS-1, and TXNIP. Activated p53 at moderate levels cooperates with active mTOR and Akt to induce senescence, whereas apoptosis is triggered by p53 at high levels when mTOR and Akt are inactivated. Remarkably, both the level and posttranslational modifications of p53 regulate the cell fate (Figure S7). The current theoretical framework could be exploited to further investigate the cellular response to energy stress.

## Data Availability

The original data are available from the corresponding author upon request.

## Supplementary Materials

The following supporting information can be downloaded at: https://www.mdpi.com/article/10.3390/ijms232314945/s1.
