# Supplementary material for: Coordination of the AMPK, Akt, mTOR, and p53 Pathways under Glucose Starvation
Source: Int J Mol Sci. 2022 Nov 29;23(23):14945. doi: 10.3390/ijms232314945 (PMC9741397; doi:10.3390/ijms232314945)
Supplement: Supplementary file 1 [file ijms-23-14945-s001.zip › ZhouY-SI.pdf]

Supplementary Materials

This study focuses on the cellular response to glucose starvation in the presence of enough growth factors and amino acids in the medium. We proposed a network model composed of five modules, characterizing glucose uptake and the AMPK, PI3K/Akt, mTOR, and p53 signaling. Generally speaking, the PI3K/Akt pathway signals the availability of growth factors and stimulates glucose uptake to support mTOR-dependent cell growth and division, while the AMPK pathway signals the lack of energy and triggers p53-dependent stress responses. The dynamics of the concentrations of species are governed by ordinary differential equations. This Supplementary Material is organized as follows. Details of model construction are first presented. The initial values of variables and parameter values are listed in Tables S1 and S2, respectively. Finally, seven supplemental figures are displayed.

Model construction and ordinary differential equations for the model network

Glucose uptake. The cell cycle is an ordered and directional process that is stimulated by growth factors. Meanwhile, kinds of nutrients (such as amino acids and glucose) are taken up to support cell proliferation. The uptake and metabolism of glucose is important for the production of ATP. Glucose uptake is stimulated by enhancing the translocation of glucose transporter GLUT1 from its storage vesicles to the plasma membrane, which is modulated by Akt and Thioredoxin-interacting protein (TXNIP). Activated Akt ($\mathrm{Akt_p}$) can promote GLUT1 transporter activity and recycling of internalized GLUT1 [1], whereas TXNIP represses glucose uptake by binding to GLUT1 and inducing its internalization [2]. These effects are described by the law of mass action: besides the basal translocation, the rate of $\mathrm{GLUT1_c}$ ($\mathrm{GLUT1_m}$) translocation is proportional to $[\mathrm{Akt_p}]$ ([TXNIP]). GLUT1 expression is also regulated by p53 and mTORC1. Activated p53 ($\mathrm{p53_p}$) binds the *glut1* promoter to repress its transcription [3], which is depicted by Hill function. Activated mTORC1 (mTOR^*) phosphorylates and activates S6K, enhancing the production of GLUT1, which is characterized by Michaelis-Menten dynamics [4]. The phosphorylation and dephosphorylation of TXNIP are enzyme-catalyzed reactions, following the Michaelis-Menten dynamics. The degradation rate of each species is proportional to its concentration. The equations for this module are presented as follows:

$$\frac{d\,[\mathrm{Glucose_i}]}{dt} = k_{\mathrm{gluin}} \frac{C_{\mathrm{g}}}{C_{\mathrm{g}} + K_{\mathrm{gluin}}} [\mathrm{GLUT1_m}] - k_{\mathrm{cata}}\, [\mathrm{Glucose_i}]\, [\mathrm{ADP}] - k_{\mathrm{dglucose}}\, [\mathrm{Glucose_i}] \tag{S1}$$

$$\frac{d\,[\mathrm{GLUT1_m}]}{dt} = \left(k_{\mathrm{sglutm}} + k_{\mathrm{aglutm}}\,[\mathrm{Akt_p}]\right)[\mathrm{GLUT1_c}] - \left(k_{\mathrm{dglutm}} + k_{\mathrm{Tglutm}}[\mathrm{TXNIP}]\right)[\mathrm{GLUT1_m}] \tag{S2}$$

$$\frac{d\,[\mathrm{GLUT1_c}]}{dt} = k_{\mathrm{sglut}} - \left(k_{\mathrm{sglutm}} + k_{\mathrm{aglutm}}\,[\mathrm{Akt_p}]\right)[\mathrm{GLUT1_c}] + \left(k_{\mathrm{dglutm}} + k_{\mathrm{Tglutm}}\,[\mathrm{TXNIP}]\right)[\mathrm{GLUT1_m}] + k_{\mathrm{rglut}}\frac{1}{1+\frac{[\mathrm{p53_p}]^4}{K_{\mathrm{rglut}}^4}} + k_{\mathrm{aglut}}\frac{[\mathrm{mTOR^*}]}{[\mathrm{mTOR^*}]+K_{\mathrm{aglut}}} - k_{\mathrm{dglut}}\,[\mathrm{GLUT1_c}] \tag{S3}$$

$$\frac{d[\mathrm{TXNIP}]}{dt} = k_{\mathrm{sTXNIP}} - k_{\mathrm{dTXNIP}}\left([\mathrm{TXNIP}] - [\mathrm{TXNIP_p}]\right) - k_{\mathrm{dTXNIP_p}}\,[\mathrm{TXNIP_p}] \tag{S4}$$

$$\frac{d\,[\mathrm{TXNIP_p}]}{dt} = k_{\mathrm{pTXNIP}}\,[\mathrm{AMPK_p}]\,\frac{([\mathrm{TXNIP}] - [\mathrm{TXNIP_p}])}{([\mathrm{TXNIP}] - [\mathrm{TXNIP_p}]) + K_{\mathrm{pTXNIP}}} - k_{\mathrm{dpTXNIP}}\frac{[\mathrm{TXNIP_p}]}{[\mathrm{TXNIP_p}] + K_{\mathrm{dpTXNIP}}} - k_{\mathrm{dTXNIP_p}}[\mathrm{TXNIP_p}] \tag{S5}$$

$[\mathrm{GLUT1_m}]$ is the level of GLUT1 that is fused with the plasma membrane, while $[\mathrm{GLUT1_c}]$ is that in the cytoplasm. C_{g} is the level of extracellular glucose and the input to the system.

Sensor of cellular energy status. Once into the cytoplasm, glucose is processed through glycolysis or oxidative phosphorylation, with ADP converted to ATP; meanwhile, the hydrolysis of ATP into ADP provides energy that supports various physiological processes. There exists the reaction 2ADP $\rightleftharpoons$ ATP + AMP[5], i.e., phosphates exchange between ATP and ADP catalyzed by the adenylate kinase. The total level of ATP, ADP and AMP is constant and is denoted by $\mathrm{AXP_t}$. As a heterotrimeric complex, AMPK is composed of the catalytic α subunit and two regulatory β and γ subunits. Active p53 triggers the induction of AMPKβ [6], which is characterized by Hill function. The activity of AMPK is regulated by phosphorylation on Thr172 in the α subunit, catalyzed by LKB1. Binding of AMP and ADP to AMPKγ protects it from dephosphorylation [7, 8]. Moreover, $\mathrm{Akt_p}$ negatively regulates AMPK activation either by suppressing its phosphorylation on Thr172 or by phosphorylating Ser485/491 of the α subunit [9]. The phosphorylation and dephosphorylation of AMPK are characterized by the Michaelis-Menten kinetics. Activated AMPK inhibits ATP-consuming processes. The equations for this module are presented in the following:

$$\frac{d[\mathrm{ATP}]}{dt} = k_{\mathrm{cata}}\,[\mathrm{Glucose_i}]\,[\mathrm{ADP}] - k_{\mathrm{ana}}\frac{[\mathrm{ATP}]}{1+\frac{[\mathrm{AMPK_p}]}{J_{\mathrm{ana}}}} + k_{\mathrm{amp}}[\mathrm{ADP}]^2 - d_{\mathrm{amp}}[\mathrm{AMP}][\mathrm{ATP}] \tag{S6}$$

$$\frac{d[\mathrm{AMP}]}{dt} = k_{\mathrm{amp}}[\mathrm{ADP}]^2 - d_{\mathrm{amp}}[\mathrm{AMP}][\mathrm{ATP}] \tag{S7}$$

$$[\mathrm{ADP}] = \mathrm{AXP_t} - [\mathrm{AMP}] - \mathrm{ATP} \tag{S8}$$

$$\frac{d[\mathrm{AMPK}]}{dt} = k_{\mathrm{sampk}} + k_{\mathrm{ampk}}\frac{[\mathrm{p53_p}]^4}{[\mathrm{p53_p}]^4 + K_{\mathrm{ampk}}^4} - k_{\mathrm{dampk}}[\mathrm{AMPK}] \tag{S9}$$

$$\frac{d\,[\mathrm{AMPK_p}]}{dt} = k_{\mathrm{pampk}}\frac{([\mathrm{AMPK}] - [\mathrm{AMPK_p}])}{([\mathrm{AMPK}] - [\mathrm{AMPK_p}]) + K_{\mathrm{pampk}}}\frac{1}{1+\frac{[\mathrm{Akt_p}]}{K_{\mathrm{aktampk}}}} - k_{\mathrm{dampk}}\,[\mathrm{AMPK_p}]$$

$$-k_{\mathrm{dampk}}\frac{[\mathrm{AMPK_p}]}{[\mathrm{AMPK_p}]+K_{\mathrm{dampk}}}\frac{1}{1+\frac{[\mathrm{AMP}]}{K_{\mathrm{amp}}}+\frac{[\mathrm{ADP}]}{K_{\mathrm{adp}}}} \tag{S10}$$

The p53 pathway. As a kinase, $\mathrm{AMPK_p}$ activates p53 by phosphorylating it on Ser15 [10, 11, 12]. As $\mathrm{p53_p}$ induces the expression of AMPKγ, a positive feedback loop exists between $\mathrm{p53_p}$ and AMPK [13, 14]. $\mathrm{p53_p}$ induces the production of Mdm2, which in turn targets p53 for degradation. Only nuclear p53 is considered here, while three forms of Mdm2 are included, namely $\mathrm{Mdm2_c}$ (cytoplasmic dephosphorylated Mdm2), $\mathrm{Mdm2_{cp}}$ (cytoplasmic phosphorylated Mdm2), and $\mathrm{Mdm2_n}$ (nuclear Mdm2). $\mathrm{Mdm2_c}$ is phosphorylated by Akt, and $\mathrm{Mdm2_{cp}}$ enters the nucleus to degrade p53. The phosphorylation and dephosphorylation of Mdm2 and p53 are represented by the Michaelis-Menten kinetics. As the ubiquitination of nuclear p53 by $\mathrm{Mdm2_n}$ is an enzyme-catalyzed reaction, it is also represented by the Michaelis-Menten kinetics. The Mdm2-dependent degradation rate of $\mathrm{p53_p}$ is much smaller than that of dephosphorylated p53.

As a transcription factor, p53 selectively induces the expression of target genes, which depends on many factors such as its co-factors and posttranslational modifications. Here, $\mathrm{p53_p}$ is divided into p53-arrester and p53-killer based on its different phosphorylation status. For simplicity, p53-arrester is primarily phosphorylated on Ser15, while p53-killer is further phosphorylated on Ser46. The conversion between p53-arrester and p53-killer is controlled by p53DINP1 [15], which is induced by both p53-arrester and p53-killer. The expression of p53-targeted genes is characterized by Hill function. The equations for this module are presented as follows:

$$\begin{aligned}\frac{d[\mathrm{p53}]}{dt} =& k_{\mathrm{sp53}} - k_{\mathrm{dp53_n}}[\mathrm{Mdm2_n}]\frac{([\mathrm{p53}]-[\mathrm{p53_p}])}{([\mathrm{p53}]-[\mathrm{p53_p}])+K_{\mathrm{dp53_n}}} \\ &-\frac{k_{\mathrm{dp53_p}}[\mathrm{Mdm2_n}][\mathrm{p53_p}]}{[\mathrm{p53_p}]+K_{\mathrm{dp53_p}}} - k_{\mathrm{dp53}}[\mathrm{p53}] \end{aligned} \tag{S11}$$

$$\begin{aligned}\frac{d[\mathrm{p53_p}]}{dt} =& k_{\mathrm{pp53}}[\mathrm{AMPK_p}]\frac{([\mathrm{p53}]-[\mathrm{p53_p}])}{([\mathrm{p53}]-[\mathrm{p53_p}])+K_{\mathrm{pp53}}} \\ &-(k_{\mathrm{dpp53mtor}}[\mathrm{mTOR^*}]+k_{\mathrm{dpp53}})\frac{[\mathrm{p53_p}]}{[\mathrm{p53_p}]+K_{\mathrm{dpp53}}} \\ &-k_{\mathrm{dp53_p}}[\mathrm{Mdm2_n}]\frac{[\mathrm{p53_p}]}{[\mathrm{p53_p}]+K_{\mathrm{dp53_p}}} - k_{\mathrm{dp53}}[\mathrm{p53_p}] \end{aligned} \tag{S12}$$

$$\begin{aligned}\frac{d[\mathrm{Mdm2_c}]}{dt} =& k_{\mathrm{smdm2}} + k_{\mathrm{mdm2}}\frac{[\mathrm{p53_p}]^4}{[\mathrm{p53_p}]^4+K_{\mathrm{mdm2}}^4} - k_{\mathrm{dmdm2}}[\mathrm{Mdm2_c}] \\ &-k_{\mathrm{pmdm2}}[\mathrm{Akt_p}]\frac{[\mathrm{Mdm2_c}]}{[\mathrm{Mdm2_c}]+K_{\mathrm{pmdm2}}} + k_{\mathrm{dpmdm2}}\frac{[\mathrm{Mdm2_{cp}}]}{[\mathrm{Mdm2_{cp}}]+K_{\mathrm{dpmdm2}}} \end{aligned} \tag{S13}$$

$$\begin{aligned}\frac{d[\mathrm{Mdm2_{cp}}]}{dt} =& k_{\mathrm{pmdm2}}[\mathrm{Akt_p}]\frac{[\mathrm{Mdm2_c}]}{[\mathrm{Mdm2_c}]+K_{\mathrm{pmdm2}}} - k_{\mathrm{dpmdm2}}\frac{[\mathrm{Mdm2_{cp}}]}{[\mathrm{Mdm2_{cp}}]+K_{\mathrm{dpmdm2}}} \\ &-k_{\mathrm{in}}[\mathrm{Mdm2_{cp}}] + k_{\mathrm{out}}[\mathrm{Mdm2_n}] - k_{\mathrm{dmdm2}}[\mathrm{Mdm2_{cp}}] \end{aligned} \tag{S14}$$

$$\frac{d[\mathrm{Mdm2_n}]}{dt} = k_{\mathrm{in}}[\mathrm{Mdm2_{cp}}] - k_{\mathrm{out}}[\mathrm{Mdm2_n}] - k_{\mathrm{dmdm2}}[\mathrm{Mdm2_n}] \tag{S15}$$

$$\frac{d[\mathrm{p53-killer}]}{dt} = k_{\mathrm{p46}}[\mathrm{p53DINP1}]\frac{[\mathrm{p53-arrester}]}{[\mathrm{p53-arrester}]+K_{\mathrm{p46}}} - k_{\mathrm{dp46}}\frac{[\mathrm{p53-killer}]}{[\mathrm{p53-killer}]+K_{\mathrm{dp46}}} \tag{S16}$$

$$[\mathrm{p53-arrester}] = [\mathrm{p53_p}] - [\mathrm{p53-killer}] \tag{S17}$$

$$\frac{d[\mathrm{p53DINP1}]}{dt} = k_{\mathrm{sdipn10}} + k_{\mathrm{dinp11}} \frac{[\mathrm{p53-arrester}]^4}{[\mathrm{p53-arrester}]^4 + K_{\mathrm{dinp11}}^4} + k_{\mathrm{dinp12}} \frac{[\mathrm{p53-killer}]^4}{[\mathrm{p53-killer}]^4 + K_{\mathrm{dinp12}}^4} - k_{\mathrm{ddinp1}}[\mathrm{p53DINP1}] \tag{S18}$$

$$\frac{d[\mathrm{p21}]}{dt} = k_{\mathrm{sp21}} + k_{\mathrm{p21}} \frac{[\mathrm{p53-arrester}]^4}{[\mathrm{p53-arrester}]^4 + K_{\mathrm{p21}}^4} - k_{\mathrm{dp21}}[\mathrm{p21}] \tag{S19}$$

$$\frac{d[\mathrm{PUMA}]}{dt} = k_{\mathrm{spuma}} + k_{\mathrm{puma}} \frac{[\mathrm{p53-killer}]^4}{[\mathrm{p53-killer}]^4 + K_{\mathrm{puma}}^4} - k_{\mathrm{dpuma}}[\mathrm{PUMA}] \tag{S20}$$

$$\frac{d[\mathrm{PTEN}]}{dt} = k_{\mathrm{spten}} + k_{\mathrm{pten}} \frac{[\mathrm{p53-killer}]^4}{[\mathrm{p53-killer}]^4 + K_{\mathrm{pten}}^4} - k_{\mathrm{dpten}}[\mathrm{PTEN}] \tag{S21}$$

The Akt pathway. Akt is activated by phosphorylation on Thr308 and Ser473 through the IRS-1/PI3K/PIP3 cascade, which is stimulated by insulin or insulin-like growth factors [16]. As we focus on the cellular response to energy stress, it is assumed that there are enough growth factors in the medium. Given continued growth factors availability, PIP3 is produced, promoting the phosphorylation of Akt. By contrast, Akt is negatively controlled by $\mathrm{AMPK_p}$ because it can phosphorylate IRS-1 [17]. The lipid phosphatase PTEN also inhibits the activation of Akt by promoting the transition from PIP3 to PIP2. The conversion from PIP2 to PIP3 is also regulated by mTORC1 substrate S6K, which phosphorylates IRS-1 on multiple Serine residues. The total levels of PIP and Akt are separately assumed to be constant. The phosphorylation and dephosphorylation of PIP3 and Akt are also represented by the Michaelis-Menten kinetics. The equations for this module are presented as follows:

$$\frac{d[\mathrm{PIP3}]}{dt} = k_{\mathrm{ppip3}} \frac{[\mathrm{PIP2}]}{[\mathrm{PIP2}] + K_{\mathrm{ppip3}}} - \left(k_{\mathrm{dppip3pten}}[\mathrm{PTEN}] + k_{\mathrm{dppip3}} + k_{\mathrm{dppip3mtor}}\,[\mathrm{mTOR^*}]\right) \frac{[\mathrm{PIP3}]}{[\mathrm{PIP3}] + K_{\mathrm{dppip3}}} \tag{S22}$$

$$[\mathrm{PIP2}] = \mathrm{PIP_t} - [\mathrm{PIP3}] \tag{S23}$$

$$\frac{d\,[\mathrm{Akt_p}]}{dt} = k_{\mathrm{pakt}}[\mathrm{PIP3}] \frac{[\mathrm{Akt}]}{[\mathrm{Akt}] + K_{\mathrm{pakt}}} - \left(k_{\mathrm{dpakt}}\,[\mathrm{AMPK_p}]\right) \frac{[\mathrm{Akt_p}]}{[\mathrm{Akt_p}] + K_{\mathrm{dpakt}}} \tag{S24}$$

$$[\mathrm{Akt}] = \mathrm{Akt_t} - [\mathrm{Akt_p}] \tag{S25}$$

The mTOR pathway. mTOR integrates the signals from nutrients, growth factors and energy status to coordinate cell growth and proliferation. mTORC1 and mTORC2 are activated in different ways and have distinct physiological functions. Only mTORC1 is considered in our model. The activity of mTORC1 is regulated by AMPK and Akt. $\mathrm{AMPK_p}$ phosphorylates and activates tuberous sclerosis 2 (TSC2), which forms a complex with its partner TSC1. The TSC1/2 complex promotes the conversion from Rheb-GTP to Rheb-GDP, thereby inhibiting mTOR activity [18, 19]. mTORC1 is activated by $\mathrm{Akt_p}$, which phosphorylates TSC2 at different sites from $\mathrm{AMPK_p}$ does, inhibiting its GAP activity toward Rheb. Additionally, mTOR dephosphorylates p53 by phosphorylating the $\alpha 4$ subunit of the PP2A phosphatase [20]. The total levels of TSC1/2, Rheb and mTOR are separately assumed to be constant. The activation and deactivation of TSC1/2, Rheb and mTOR are described by the Michaelis-Menten kinetics. The equations for this module are presented

as follows:

$$\frac{d\,[\text{TSC1/2}^*]}{dt} = k_{\text{atsc}}\,[\text{AMPK}_{\text{p}}]\,\frac{\text{TSC}_{\text{t}} - [\text{TSC1/2}^*]}{\text{TSC}_{\text{t}} - [\text{TSC1/2}^*] + K_{\text{atsc}}} - k_{\text{dtsc}}\,[\text{Akt}_{\text{p}}]\,\frac{[\text{TSC1/2}^*]}{[\text{TSC1/2}^*] + K_{\text{dtsc}}} \tag{S26}$$

$$\frac{d[\text{Rheb-GTP}]}{\text{dt}} = k_{\text{arheb}}\frac{[\text{Rheb-GDP}]}{[\text{Rheb-GDP}] + K_{\text{arheb}}} - k_{\text{drheb}}\,[\text{TSC1/2}^*]\,\frac{[\text{Rheb-GTP}]}{[\text{Rheb-GTP}] + K_{\text{drheb}}} \tag{S27}$$

$$[\text{Rheb-GDP}] = \text{Rheb}_{\text{t}} - [\text{Rheb-GTP}] \tag{S28}$$

$$\frac{d\,[\text{mTOR}^*]}{dt} = k_{\text{amtor}}\,[\text{Rheb} - \text{GTP}]\frac{[\text{mTOR}]}{[\text{mTOR}] + K_{\text{amtor}}} - k_{\text{damtor}}\,\frac{[\text{mTOR}^*]}{[\text{mTOR}^*] + K_{\text{damtor}}} \tag{S29}$$

$$[\text{mTOR}] = \text{mTOR}_{\text{t}} - [\text{mTOR}^*] \tag{S30}$$

Output of the model network. The cellular outcomes include proliferation, senescence, and apoptosis. The cell-cycle arrest is induced by p21, whereas apoptosis is triggered by PUMA. Here, persistent cell-cycle arrest together with mTOR activation is considered as the indicator of senescence, while sustained activation of PUMA is the marker of apoptosis. Constrained by available experimental data, we did not model the events following the induction of p21 and PUMA, and thus the irreversibility of senescence and apoptosis was not explored.

References

[1] H. L. Wieman, J. A. Wofford, and J. C. Rathmell, "Cytokine stimulation promotes glucose uptake via phosphatidylinositol-3 kinase/Akt regulation of Glut1 activity and trafficking," *Mol. Biol. Cell*, vol. 18, no. 4, pp. 1437–1446, 2007.

[2] N. Wu, B. Zheng, A. Shaywitz, Y. Dagon, C. Tower, G. Bellinger, C.-H. Shen, J. Wen, J. Asara, T. E. McGraw, B. B. Kahn, and L. C. Cantley, "AMPK-dependent degradation of TXNIP upon energy stress leads to enhanced glucose uptake via GLUT1," *Mol. Cell*, vol. 49, no. 6, pp. 1167–1175, 2013.

[3] F. Schwartzenberg-Bar-Yoseph, M. Armoni, and E. Karnieli, "The tumor suppressor p53 down-regulates glucose transporters GLUT1 and GLUT4 gene expression," *Cancer Res.*, vol. 64, no. 7, pp. 2627–2633, 2004.

[4] C. L. Buller, R. D. Loberg, M. H. Fan, Q. Zhu, J. L. Park, E. Vesely, K. Inoki, K. L. Guan, and F. C. Brosius III, "A GSK-3/TSC2/mTOR pathway regulates glucose uptake and GLUT1 glucose transporter expression," *Am. J. Physiol., Cell Physiol.*, vol. 295, no. 3, pp. C836–843, 2008.

[5] D. G. Hardie, F. A. Ross, and S. A. Hawley, "AMPK: a nutrient and energy sensor that maintains energy homeostasis," *Nat. Rev. Mol. Cell Biol.*, vol. 13, no. 4, pp. 251–262, 2012.

[6] Z. Feng, W. Hu, E. de Stanchina, A. K. Teresky, S. Jin, S. Lowe, and A. J. Levine, "The regulation of AMPK β1, TSC2, and PTEN expression by p53: Stress, cell and tissue specificity, and the role of these gene products in modulating the IGF-1-AKT-mTOR pathways," *Cancer Res.*, vol. 67, no. 7, pp. 3043–3053, 2007.

[7] J. S. Oakhill, Z.-P. Chen, J. W. Scott, R. Steel, L. A. Castelli, N. Ling, S. L. Macaulay, and B. E. Kemp, "β-subunit myristoylation is the gatekeeper for initiating metabolic stress sensing by AMP-activated protein kinase (AMPK)," *Proc. Natl. Acad. Sci. U.S.A.*, vol. 107, no. 45, pp. 19237–19241, 2010.

[8] B. Xiao, M. J. Sanders, E. Underwood, R. Heath, F. V. Mayer, D. Carmena, C. Jing, P. A. Walker, J. F. Eccleston, L. F. Haire, P. Saiu, S. A. Howell, R. Aasland, S. R. Martin, D. Carling, and S. J. Gamblin, "Structure of mammalian AMPK and its regulation by ADP," *Nature*, vol. 472, no. 7342, pp. 230–233, 2011.

[9] R. J. Valentine, K. A. Coughlan, N. B. Ruderman, and A. K. Saha, "Insulin inhibits AMPK activity and phosphorylates AMPK Ser485/491 through Akt in hepatocytes, myotubes and incubated rat skeletal muscle," *Arch. Biochem. Biophys.*, vol. 562, pp. 62–69, 2014.

[10] K. Imamura, T. Ogura, A. Kishimoto, M. Kaminishi, and H. Esumi, "Cell cycle regulation via p53 phosphorylation by a 5'-AMP activated protein kinase activator, 5-aminoimidazole-4-carboxamide-1-beta-d-ribofuranoside, in a human hepatocellular carcinoma cell line," *Biochem. Biophys. Res. Commun.*, vol. 287, no. 2, pp. 562–567, 2001.

[11] M. Igata, H. Motoshima, K. Tsuruzoe, K. Kojima, T. Matsumura, T. Kondo, T. Taguchi, K. Nakamaru, M. Yano, D. Kukidome, K. Matsumoto, T. Toyonaga, T. Asano, T. Nishikawa, and E. Araki, "Adenosine monophosphate-activated protein kinase suppresses vascular smooth muscle cell proliferation through the inhibition of cell cycle progression," *Circ. Res.*, vol. 97, no. 8, pp. 837–844, 2005.

[12] R. G. Jones, D. R. Plas, S. Kubek, M. Buzzai, J. Mu, Y. Xu, M. J. Birnbaum, and C. B. Thompson, "AMP-activated protein kinase induces a p53-dependent metabolic checkpoint," *Mol. Cell*, vol. 18, no. 3, pp. 283–293, 2005.

[13] M. Bazuine, K. G. Stenkula, M. Cam, M. Arroyo, and S. W. Cushman, "Guardian of corpulence: a hypothesis on p53 signaling in the fat cell," *Clin. Lipidol.*, vol. 4, no. 2, pp. 231–243, 2009.

[14] A. Efeyan and D. M. Sabatini, "mTOR and cancer: many loops in one pathway," *Curr. Opin. Cell Biol.*, vol. 22, no. 2, pp. 169–176, 2010.

[15] S. Okamura, H. Arakawa, T. Tanaka, H. Nakanishi, C. C. Ng, Y. Taya, M. Monden, and Y. Nakamura, "p53DINP1, a p53-inducible gene, regulates p53-dependent apoptosis," *Mol. Cell*, vol. 8, no. 1, pp. 85–94, 2001.

[16] T. F. Franke, C. P. Hornik, L. Segev, G. A. Shostak, and C. Sugimoto, "PI3K/Akt and apoptosis: size matters," *Oncogene*, vol. 22, no. 56, pp. 8983–8998, 2003.

[17] A. Tzatsos and P. N. Tsichlis, "Energy depletion inhibits phosphatidylinositol 3-kinase/Akt signaling and induces apoptosis via AMP-activated protein kinase-dependent phosphorylation of IRS-1 at Ser-794," *J. Biol. Chem.*, vol. 282, no. 25, pp. 18069–18082, 2007.

[18] K. Inoki, T. Zhu, and K. L. Guan, "TSC2 mediates cellular energy response to control cell growth and survival," *Cell*, vol. 115, no. 5, pp. 577–590, 2003.

[19] R. J. Shaw, N. Bardeesy, B. D. Manning, L. Lopez, M. Kosmatka, R. A. DePinho, and L. C. Cantley, "The LKB1 tumor suppressor negatively regulates mTOR signaling," *Cancer Cell*, vol. 6, no. 1, pp. 91–99, 2004.

[20] M. Kong, C. J. Fox, J. Mu, L. Solt, A. Xu, R. M. Cinalli, M. J. Birnbaum, T. Lindsten, and C. B. Thompson, "The PP2A-associated protein $\alpha 4$ is an essential inhibitor of apoptosis," *Science*, vol. 306, no. 5696, pp. 695–698, 2004.

Supplemental Table S1: Variables of the model

Variable	Description	Initial value
[$\mathrm{Glucose_i}$]	Concentration of intracellular glucose	9.5322
[$\mathrm{GLUT_m}$]	Concentration of GLUT1 fused with plasma membrane	0.3883
[$\mathrm{GLUT_c}$]	Concentration of cytoplasmic GLUT1	0.6764
[TXNIP]	Concentration of TXNIP	0.8324
[$\mathrm{TXNIP_p}$]	Concentration of phosphorylated TXNIP	0.1308
[ATP]	Concentration of ATP	9.4967
[AMP]	Concentration of AMP	0.0242
[AMPK]	Concentration of AMPK	0.1000
[$\mathrm{AMPK_p}$]	Concentration of active AMPK	0.0020
[p53]	Concentration of p53	0.6006
[$\mathrm{p53_p}$]	Concentration of active p53	0.0214
[$\mathrm{Mdm2_c}$]	Concentration of cytoplasmic dephosphorylated Mdm2	0.2175
[$\mathrm{Mdm2_{cp}}$]	Concentration of cytoplasmic phosphorylated Mdm2	0.4980
[$\mathrm{Mdm2_n}$]	Concentration of nuclear Mdm2	0.2845
[p53-killer]	Concentration of p53-killer	0.0016
[p53DINP1]	Concentration of p53DINP1	0.0165
[p21]	Concentration of p21	0.1000
[PTEN]	Concentration of PTEN	0.1003
[PUMA]	Concentration of PUMA	0.1000
[PIP3]	Concentration of PIP3	0.8858
[$\mathrm{Akt_p}$]	Concentration of active Akt	1.0000
[TSC1/2*]	Concentration of TSC1/2*	0.0000
[Rheb-GTP]	Concentration of Rheb-GTP	1.0000
[mTOR*]	Concentration of mTOR*	0.9738

Supplemental Table S2: Parameters of the model

Rate Constant	Description	Value
k_{cata}	Rate of catabolism	2 min^{-1}
k_{gluin}	Rate constant of glucose uptake	50 min^{-1}
K_{gluin}	Michaelis constant of glucose uptake	1.3 mM
k_{dglucose}	Dilution rate of intracellular glucose	1 min^{-1}
k_{sglutm}	Basal rate of GLUT1 translocation to the membrane	0.003 min^{-1}
k_{aglutm}	Rate constant of Akt-activated GLUT1 translocation to the membrane	0.01 min^{-1}
k_{dglutm}	Basal rate of GLUT1 translocation to the cytoplasm	0.006 min^{-1}
k_{Tglutm}	Rate constant of TXNIP-promoted GLUT1 translocation to the cytoplasm	0.02 min^{-1}
k_{sglut}	Basal production rate of GLUT1	0.001 min^{-1}
k_{dglut}	Basal degradation rate of GLUT1	0.01 min^{-1}
k_{aglut}	Maximum rate of mTOR-regulated GLUT1 expression	0.001 min^{-1}
K_{aglut}	Michaelis constant of mTOR-regulated GLUT1 production	0.3
k_{rglut}	Maximum rate of p53-regulated GLUT1 expression	0.005 min^{-1}
K_{rglut}	Michaelis constant of p53 repression	0.3
k_{sTXNIP}	Basal production rate of TXNIP	0.0201 min^{-1}
k_{dTXNIP}	Degradation rate of TXNIP	0.01 min^{-1}
k_{pTXNIP}	Phosphorylation rate of TXNIP	1.2 min^{-1}
K_{pTXNIP}	Michaelis constant of TXNIP phosphorylation	0.5
k_{dpTXNIP}	Dephosphorylation rate of TXNIP	0.5 min^{-1}
K_{dpTXNIP}	Michaelis constant of TXNIP dephosphorylation	0.5
$k_{\text{dTXNIP}_\text{p}}$	Degradation rate of TXNIP_p	0.3 min^{-1}
AXP_t	The total concentration of ATP, ADP and AMP	10
k_{ana}	Rate of anabolism	1 min^{-1}
J_{ana}	Michaelis constant of AMPK-dependent inhibition of anabolism	0.05
k_{amp}	Rate of forward adenylate kinase reaction	1 min^{-1}
d_{amp}	Rate of reverse adenylate kinase reaction	1 min^{-1}
k_{sampk}	Basal production rate of AMPK	0.0005 min^{-1}
k_{ampk}	p53-dependent production rate of AMPK	0.01 min^{-1}

Continued on next page

Supplemental Table S2: Parameters of the model (Continued)

Rate Constant	Description	Value
K_{ampk}	Michaelis constant of p53-induced AMPK production	0.4
k_{dampk}	Degradation rate of AMPK	0.01 min^{-1}
K_{amp}	Michaelis constant of AMP-dependent inhibition of AMPK deactivation	0.15
K_{adp}	Michaelis constant of ADP-dependent inhibition of AMPK deactivation	0.2
k_{pampk}	Phosphorylation rate of AMPK	0.3 min^{-1}
k_{dpampk}	Dephosphorylation rate of AMPK	2 min^{-1}
K_{pampk}	Michaelis constant of AMPK phosphorylation	0.2
K_{dpampk}	Michaelis constant of AMPK dephosphorylation	0.1
K_{aktampk}	Michaelis constant of Akt-dependent inhibition of AMPK activation	0.5
k_{sp53}	Basal production rate of p53	0.2 min^{-1}
k_{dp53}	Basal degradation rate of p53	0.05 min^{-1}
k_{dp53n}	Mdm2-dependent degradation rate of p53	0.7 min^{-1}
$K_{\mathrm{dp53_n}}$	Michaelis constant of Mdm2-dependent p53 degradation	0.1
k_{pp53}	AMPK-dependent activation rate of p53	4.2 min^{-1}
K_{pp53}	Michaelis constant of AMPK-dependent p53 acitvation	0.28
k_{dpp53}	Basal deactivation rate of p53	0.15 min^{-1}
$k_{\mathrm{dpp53mtor}}$	mTOR-dependent deactivation rate of p53	0.15 min^{-1}
K_{dpp53}	Michaelis constant of p53 deactivation	0.7
$k_{\mathrm{dp53_p}}$	Mdm2-dependent degradation rate of $\mathrm{p53_p}$	0.01 min^{-1}
$K_{\mathrm{dp53_p}}$	Michaelis constant of Mdm2-dependent $\mathrm{p53_p}$ degradation	0.5
k_{smdm2}	Basal production rate of Mdm2	0.01 min^{-1}
k_{mdm2}	p53-dependent production rate of Mdm2	0.008 min^{-1}
K_{mdm2}	Michaelis constant of p53-dependent Mdm2 production	1
k_{dmdm2}	Degradation rate of Mdm2	0.01 min^{-1}
k_{pmdm2}	Akt-dependent phosphorylation rate of $\mathrm{Mdm2_c}$	2 min^{-1}
K_{pmdm2}	Michaelis constant of Akt-dependent phosphorylation	0.3
k_{dpmdm2}	Dephosphorylation rate of $\mathrm{Mdm2_{cp}}$	1 min^{-1}
K_{dpmdm2}	Michaelis constant of dephosphorylation of $\mathrm{Mdm2_{cp}}$	0.1

Continued on next page

Supplemental Table S2: Parameters of the model (Continued)

Rate Constant	Description	Value
k_{in}	Nuclear import rate of Mdm2_{cp}	0.04 min^{-1}
k_{out}	Neclear export rate of Mdm2_{n}	0.06 min^{-1}
k_{p46}	Rate constant of p53-arrester phosphorylation	2.6 min^{-1}
K_{p46}	Michaelis constant of p53-arrester phosphorylation	0.32
k_{dp46}	Rate constant of p53-killer dephosphorylation	2 min^{-1}
K_{dp46}	Michaelis constant of p53-killer dephosphorylation	0.1
k_{sdinp10}	Basal production rate of p53DINP1	0.000014 min^{-1}
k_{dinp11}	p53-arrester induced production rate of p53DINP1	0.0018 min^{-1}
K_{dinp11}	Michaelis constant of p53-arrester dependent p53DINP1 production	0.9
k_{dinp12}	p53-killer induced production rate of p53DINP1	0.0021 min^{-1}
K_{dinp11}	Michaelis constant of p53-killer dependent p53DINP1 production	1.5
k_{ddinp1}	Degradation rate of p53DINP1	0.00084 min^{-1}
k_{sp21}	Basal production rate of p21	0.001 min^{-1}
k_{p21}	p53-arrester induced production rate of p21	0.04 min^{-1}
K_{p21}	Michaelis constant of p53-arrester dependent p21 production	0.57
k_{dp21}	Degradation rate of p21	0.01 min^{-1}
k_{spten}	Basal production rate of PTEN	0.00035 min^{-1}
k_{pten}	p53-killer induced production rate of PTEN	0.0175 min^{-1}
K_{pten}	Michaelis constant of p53-killer dependent PTEN production	0.6
k_{dpten}	Degradation rate of PTEN	0.0035 min^{-1}
k_{spuma}	Basal production rate of PUMA	0.001 min^{-1}
k_{puma}	p53-killer induced production rate of PUMA	0.03 min^{-1}
K_{puma}	Michaelis constant of p53-killer dependent PUMA production	1.5
k_{dpuma}	Degradation rate of PUMA	0.01 min^{-1}
k_{ppip3}	Phosphorylation rate of PIP2	0.13 min^{-1}
K_{ppip3}	Michaelis constant of PIP2 phosphorylation	0.2
k_{dppip3}	Basal dephosphorylation rate of PIP3	0.01 min^{-1}
$k_{\text{dppip3pten}}$	PTEN-dependent dephosphorylation rate of PIP3	0.07 min^{-1}

Continued on next page

Supplemental Table S2: Parameters of the model (Continued)

Rate Constant	Description	Value
$k_{\mathrm{dppip3mtor}}$	mTOR-dependent dephosphorylation rate of PIP3	0.042 min^{-1}
K_{dppip3}	Michaelis constant of PIP3 dephosphorylation	0.2
k_{pakt}	Phophorylation rate of Akt	0.34 min^{-1}
K_{pakt}	Michaelis constant of Akt phosphorylation	0.1
k_{dpakt}	Dephosphorylation rate of $\mathrm{Akt_p}$	0.06 min^{-1}
K_{dpakt}	Michaelis constant of $\mathrm{Akt_p}$ dephosphorylation	0.01
$\mathrm{Akt_t}$	The total concentration of Akt and $\mathrm{Akt_p}$	1
$\mathrm{PIP_t}$	The total concentration of PIP2 and PIP3	1
k_{atsc}	Activation rate of TSC	8 min^{-1}
$\mathrm{TSC_t}$	The total concentration of active and inactive TSC	1
K_{atsc}	Michaelis constant of TSC activation	0.1
k_{dtsc}	Deactivation rate of active TSC	3 min^{-1}
K_{dtsc}	Michaelis constant of TSC deactivation	0.1
k_{arheb}	Activation rate of Rheb	1 min^{-1}
$\mathrm{Rheb_t}$	The total concentration of Rheb-GTP and Rheb-GDP	1
K_{arheb}	Michaelis constant of Rheb activation	0.5
k_{drheb}	Deactivation rate of Rheb	3 min^{-1}
K_{drheb}	Michaelis constant of Rheb deactivation	0.5
k_{amtor}	Activation rate of mTORC1	4.2 min^{-1}
$\mathrm{mTOR_t}$	The total concentration of active and inactive mTORC1	1
K_{amtor}	Michaelis constant of mTORC1 activation	0.025
k_{damtor}	Deactivation rate of active mTORC1	0.6 min^{-1}
K_{damtor}	Michaelis constant of mTORC1 deactivation	0.01

Supplemental Figures

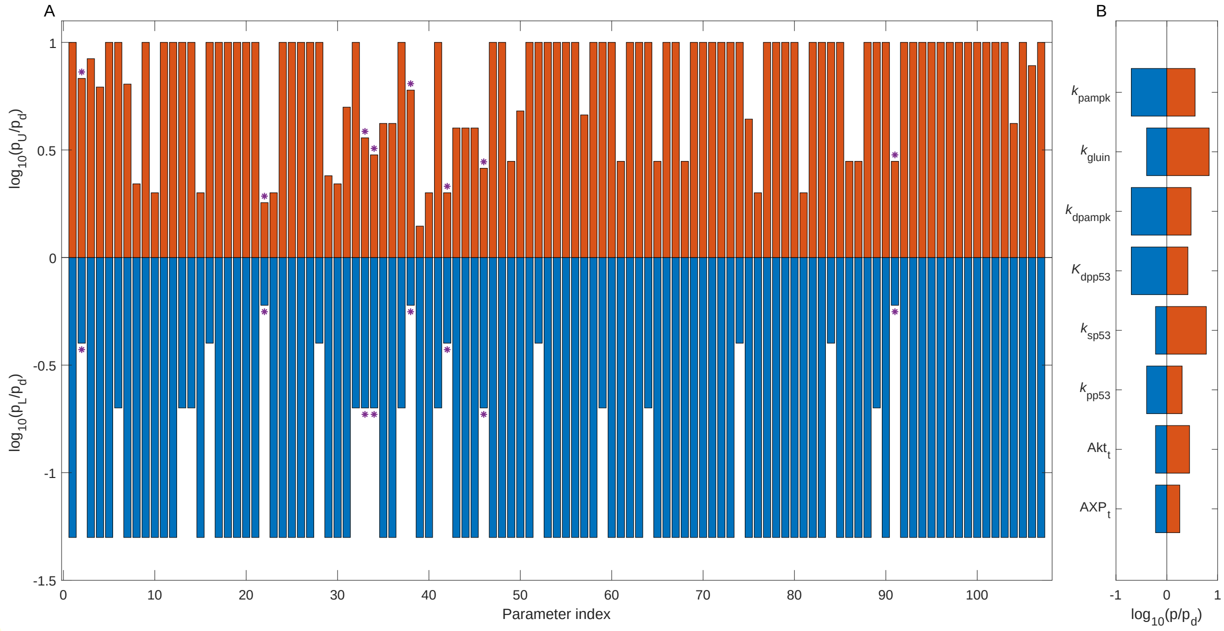

Figure S1: Parameter sensitivity analysis. (A) Base-10 logarithm of the ratio of the upper (red) or lower (blue) bound of each parameter to its default value. For each value between the two bounds, the cell can sequentially undergo proliferation, senescence and apoptosis when the glucose concentration is decreased gradually from a high level. Parameters are indexed in the order as in Supplemental Table 2 (from top to bottom). The absolute values of two ratios are summed to measure the sensitivity, and eight most sensitive parameters are marked by purple asterisk. (B) Ratios for those eight parameters marked by purple asterisk.

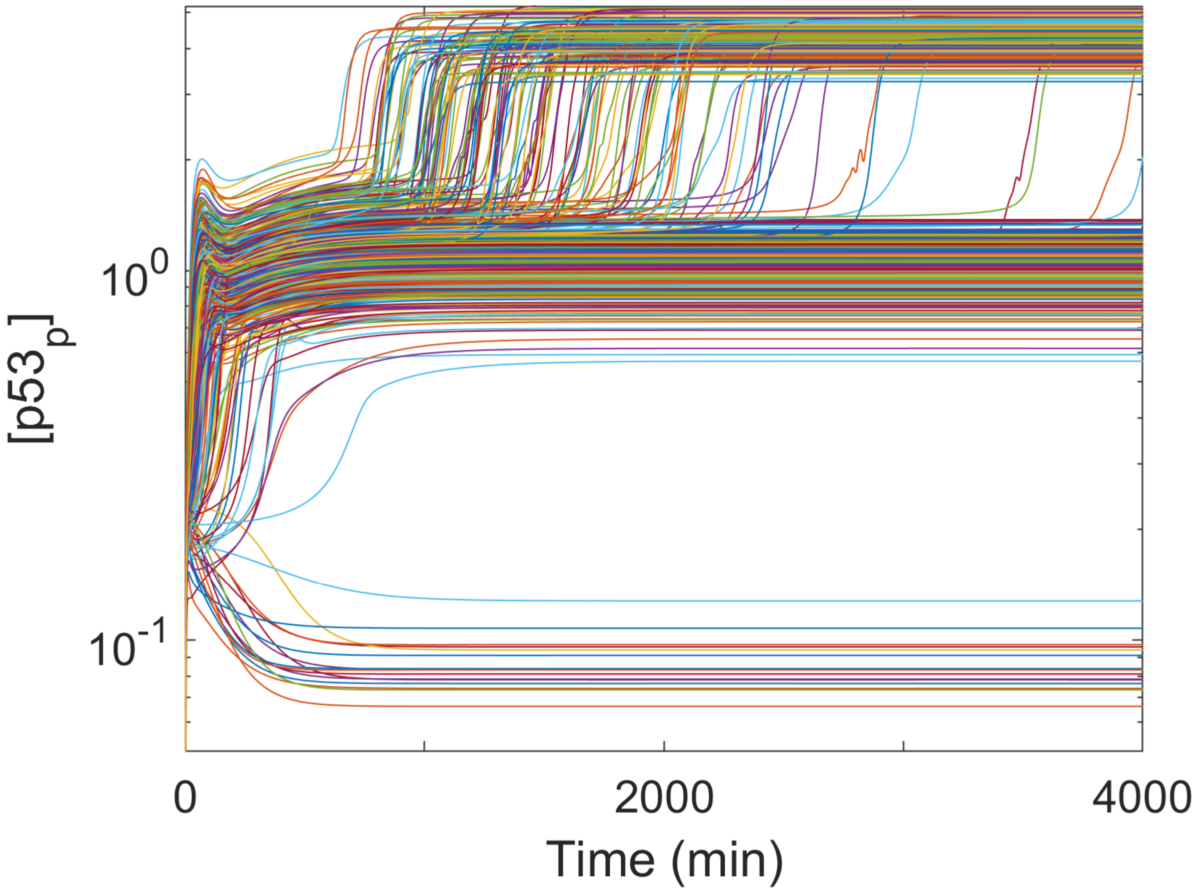

Figure S2: Time courses of the $p53_p$ level for a population of cells exposed to the same stress. Shown are 500 trajectories for $C_g = 0.2$ mM. The value of every parameter for each cell is randomly taken from a uniform distribution on the interval from 85% to 115% of its default value.

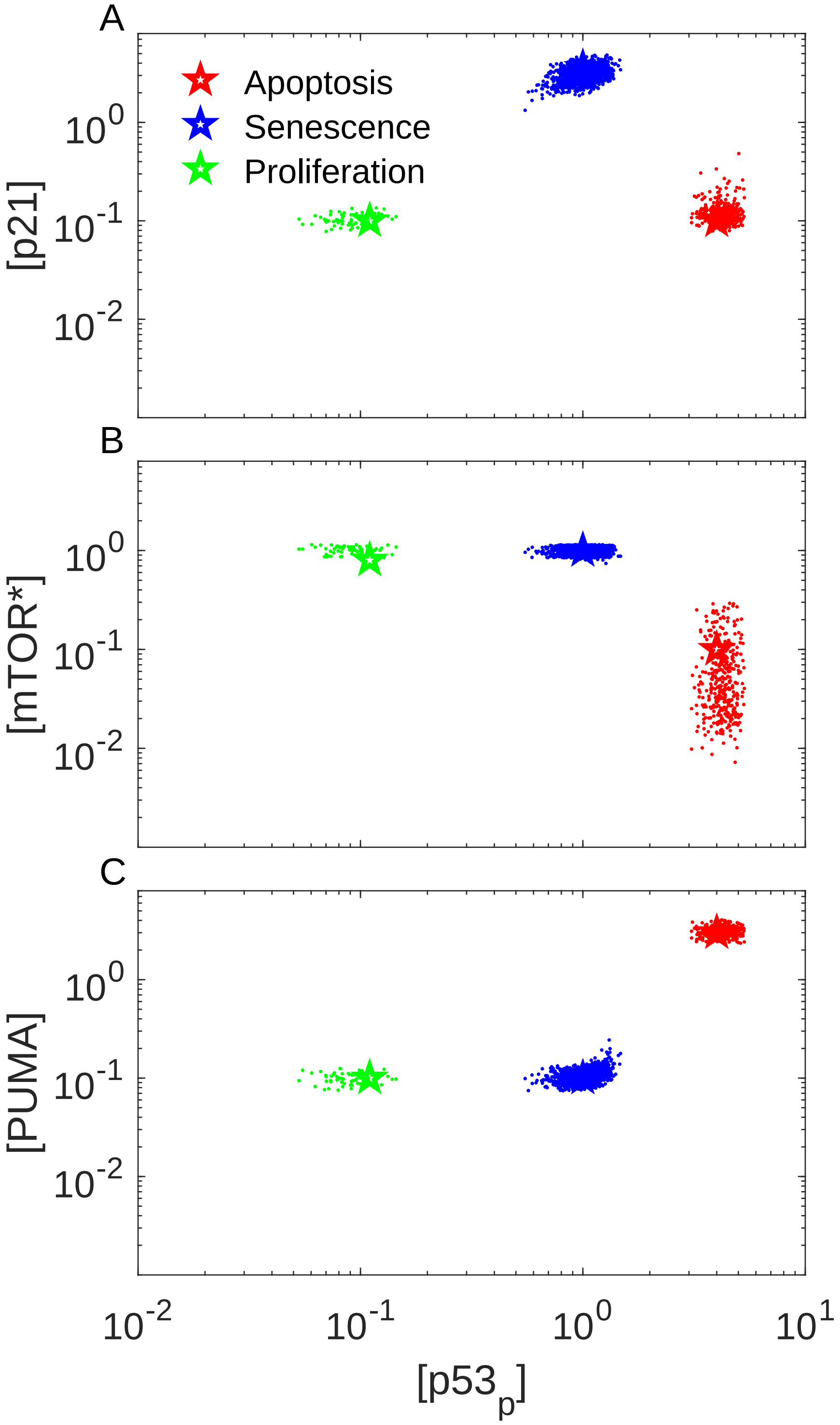

Figure S3: Displayed are the steady-state levels of p21 (A), mTOR* (B) and PUMA (C) versus the $p53_p$ level. Each dot represents the state of one cell. The same convention is used as in Fig. S2.

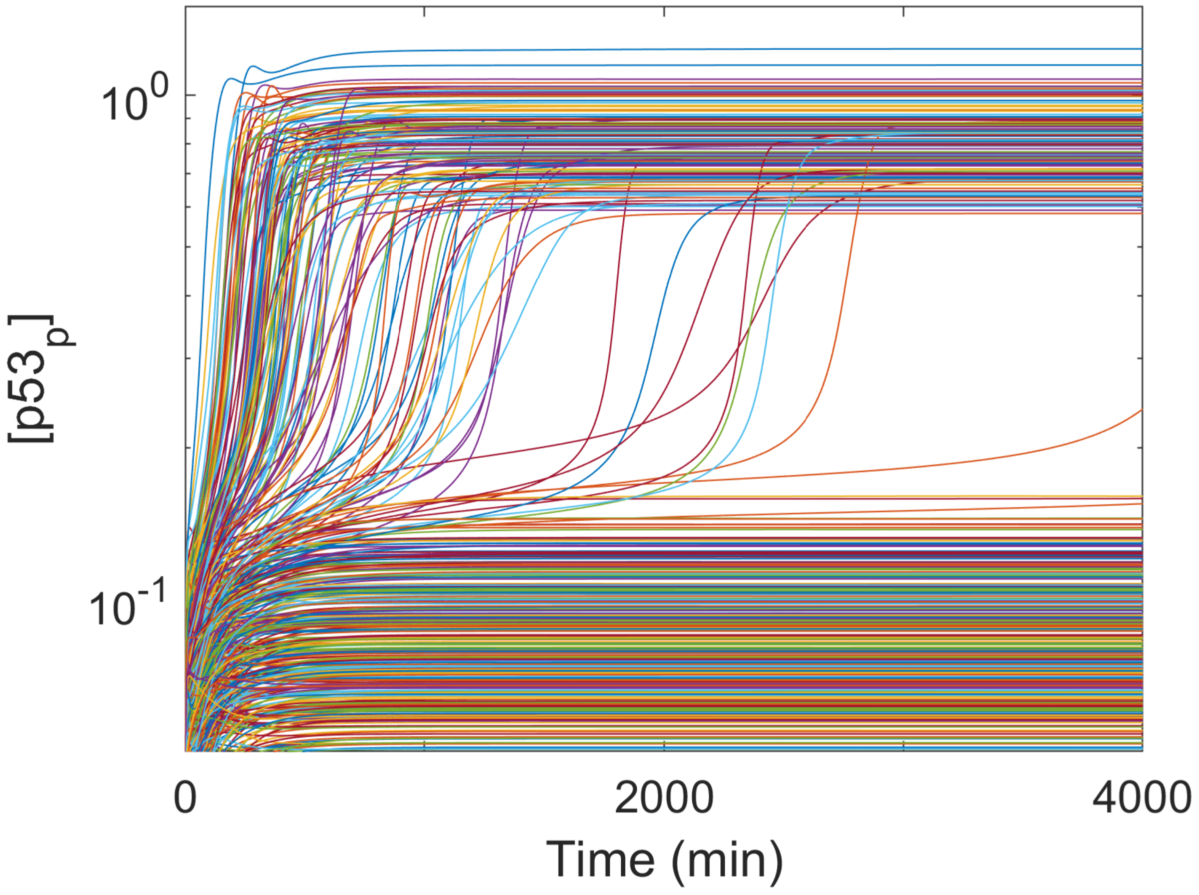

Figure S4: Time courses of the $p53_p$ level for a population of cells exposed to the same stress. Shown are 500 trajectories for $C_g = 2$ mM. The value of every parameter for each cell is randomly taken from a uniform distribution on the interval from 85% to 115% of its default value.

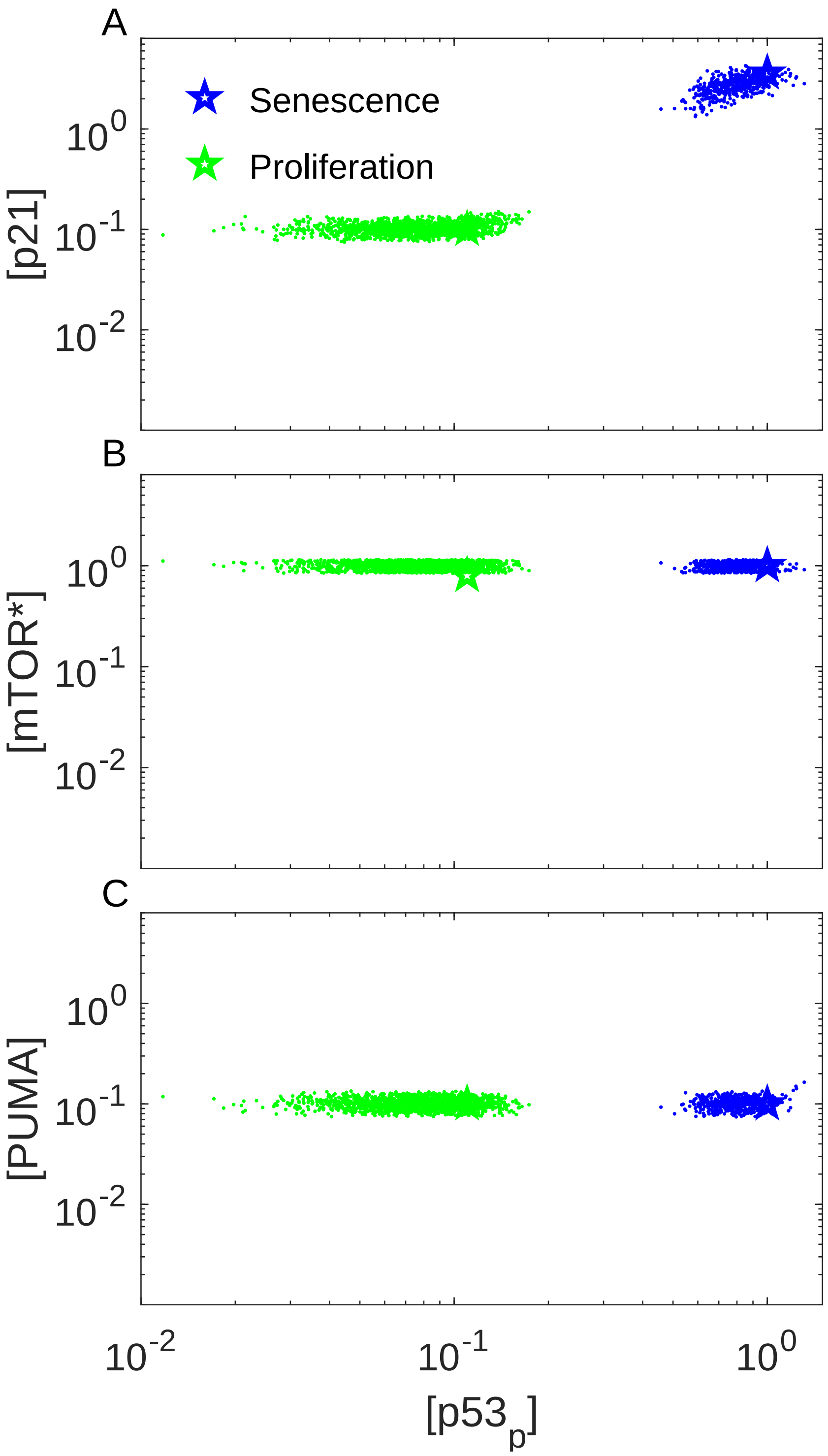

Figure S5: Displayed are the steady-state levels of p21 (A), mTOR* (B) and PUMA (C) versus the $p53_p$ level. Each dot represents the state of one cell. The same convention is used as in Fig. S4.

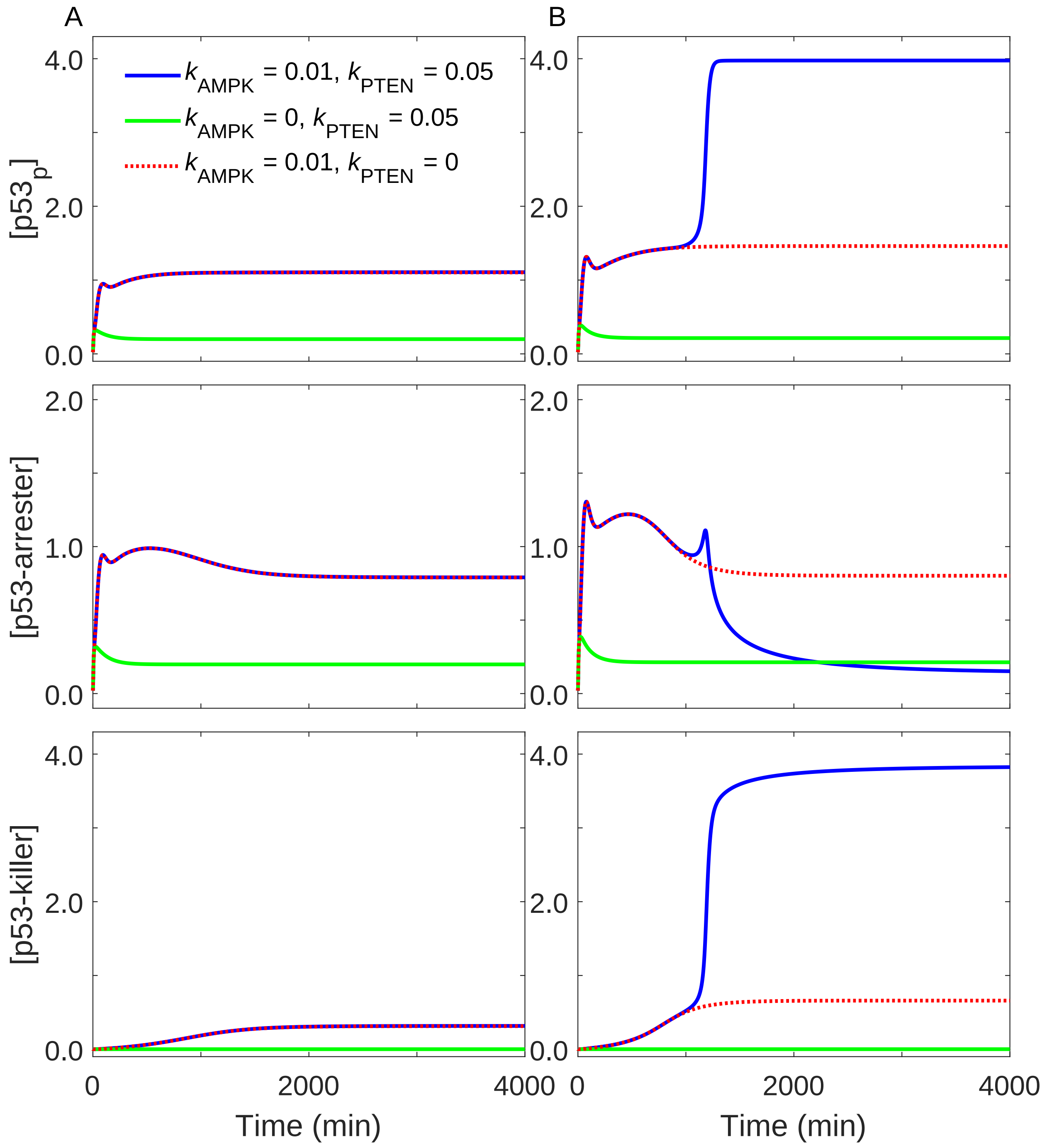

Figure S6: Role for the two positive feedback loops in cell-fate decision. Shown is temporal evolution of the levels of p53$_p$, p53-arrester, and p53-killer (from top to bottom) with the default parameter setting ($k_{AMPK} = 0.01$, $k_{PTEN} = 0.05$, *blue*), or when the p53-dependent transcription of AMPK is blocked ($k_{AMPK} = 0, k_{PTEN} = 0.05$, *green*), or when the p53-dependent transcription of PTEN is blocked ($k_{AMPK} = 0.01, k_{PTEN} = 0$, *red*). The glucose level is 0.2 mM (A) or 0.1 mM (B).

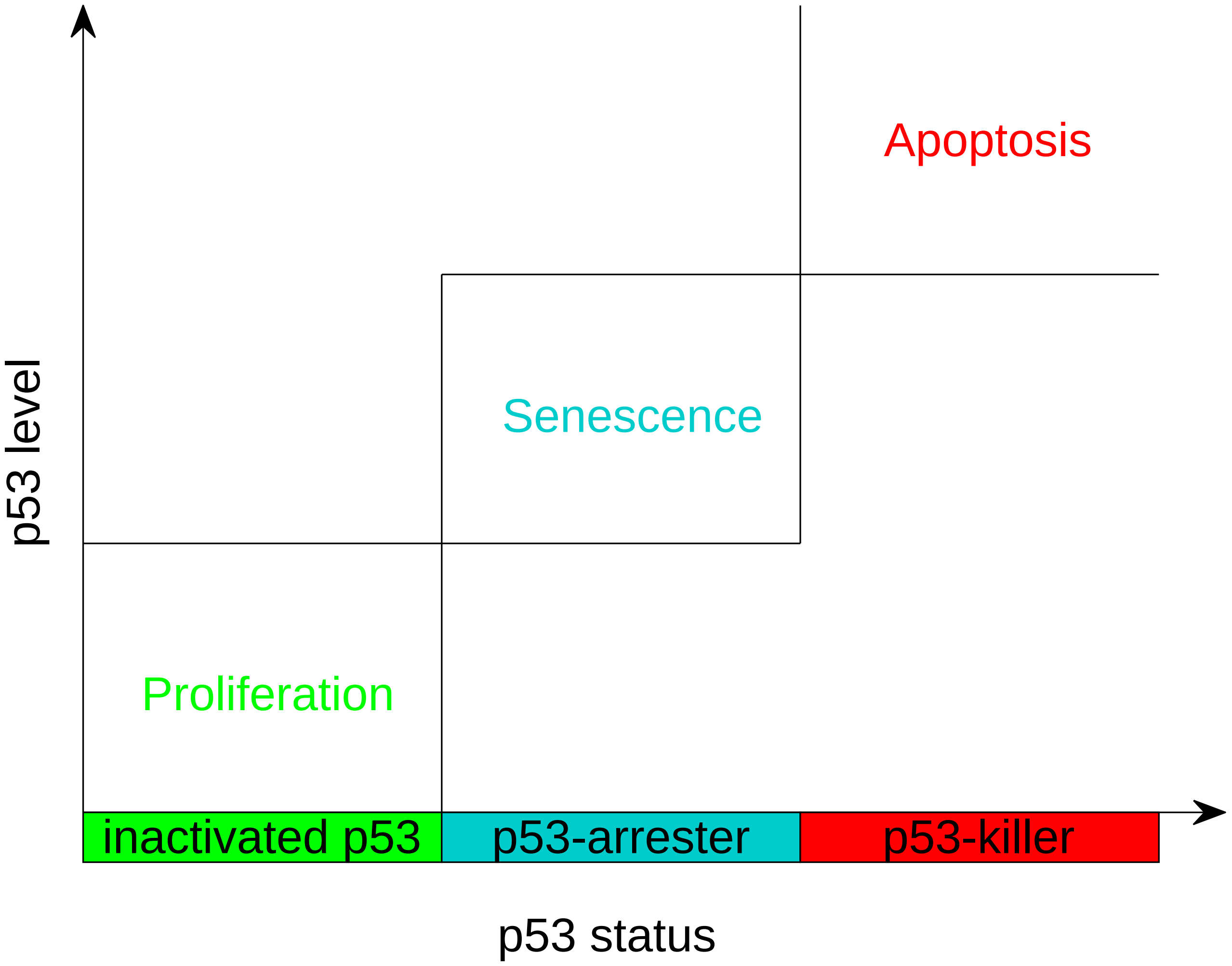

Figure S7: Schematic depiction of how the cell fate is regulated by both the level and posttranslational modifications of p53.
